# Land-locked mammalian Golgi reveals cargo transport between stable cisternae

**DOI:** 10.1038/s41467-017-00570-z

**Published:** 2017-09-05

**Authors:** Myun Hwa Dunlop, Andreas M. Ernst, Lena K. Schroeder, Derek K. Toomre, Grégory Lavieu, James E. Rothman

**Affiliations:** Department of Cell Biology, Yale School of Medicine, New Haven, CT 06511 USA

## Abstract

The Golgi is composed of a stack of *cis*, *medial*, *trans* cisternae that are biochemically distinct. The stable compartments model postulates that permanent cisternae communicate through bi-directional vesicles, while the cisternal maturation model postulates that transient cisternae biochemically mature to ensure anterograde transport. Testing either model has been constrained by the diffraction limit of light microscopy, as the cisternae are only 10–20 nm thick and closely stacked in mammalian cells. We previously described the unstacking of Golgi by the ectopic adhesion of Golgi cisternae to mitochondria. Here, we show that cargo processing and transport continue—even when individual Golgi cisternae are separated and “land-locked” between mitochondria. With the increased spatial separation of cisternae, we show using three-dimensional live imaging that *cis*-Golgi and *trans*-Golgi remain stable in their composition and size. Hence, we provide new evidence in support of the stable compartments model in mammalian cells.

## Abstract (web summary)

The different composition of Golgi cisternae gave rise to two different models for intra-Golgi traffic: one where stable cisternae communicate via vesicles and another one where cisternae biochemically mature to ensure anterograde transport. Here, the authors provide evidence in support of the stable compartments model.

## Introduction

The Golgi is composed of a stack of flattened cisternae that serves as an obligate intermediate for membrane trafficking of proteins in the secretory pathway^1^. Proteins and lipids enter from the endoplasmic reticulum (ER) into the *cis-*Golgi, where they undergo extensive modification as they traverse the Golgi, including glycosylation, sulfation, phosphorylation, lipidation, and proteolysis. Then, they leave via the *trans-*Golgi for secretion, presentation on the cell surface, or to a target endomembrane system. Over a third of proteins encoded in the human genome pass through this secretory pathway^2^. Hence, proper functioning of the Golgi is critical for many cellular activities.

The mechanism of anterograde (*cis*-to-*trans* direction) membrane traffic through the Golgi remains debated^1, 3, 4^. There are two main models, of which the first is the stable compartments model. Here, the Golgi exists as a long-lived compartment that receives and exports cargo through anterograde COPI-coated vesicles (and in some variations, transient tubular intermediates)^5–10^. Resident proteins and glycosylation enzymes are distributed across the stack in a polarized fashion, and any leaked resident enzymes would be recycled back in retrograde COPI vesicles. The second model is the cisternal progression/maturation model^1, 3^. In this model, the Golgi cisterna itself is the cargo carrier that gradually progresses/matures from *cis* to *medial* to *trans* by the uptake of retrograde COPI vesicles that deliver resident enzymes from older to younger cisternae.

Cisternal maturation was directly visualized in *S. cerevisiae*, where the fate of Golgi cisterna can be observed in living cells by confocal imaging because they exist physiologically as separate (unstacked) entities^11^. Over the course of 6–7 min, early resident proteins were observed to be replaced by late resident proteins^12, 13^. However, the same live imaging experiment has been impossible to recapitulate in mammalian cells where the Golgi cisternae are in fact stacked. Here, individual cisternae are closer to each other than the diffraction limit of light microscopy and thus cannot be resolved by conventional imaging. Super-resolution live cell imaging in three dimensions at sufficient resolution to track vesicles (<50 nm) would be required but has not yet been achieved.

But can the cisternae comprising normally stacked Golgi be forced apart in the cell without seriously impeding their function? If this were the case, it would enable experiments analogous to those pioneered in yeast to critically distinguish transport models. One approach would be to remove a key stacking protein in vivo. GRASP55 and GRASP65 were originally identified as stacking factors for their ability to restack Golgi cisternae in a cell-free system^14, 15^. Immuno-electron microscopy (EM) revealed that GRASP55 localizes to the *medial/trans-*cisternae, while GRASP65 localizes to the *cis-*cisternae, suggesting that both are required to build a polarized Golgi stack^14, 16^. The N-terminal half of both GRASPs consists of a highly conserved GRASP domain that can form homodimers^14, 16–18^. The dual anchoring of the GRASP domain by an N-terminal myristic acid and a C-terminal Golgin-binding domain restricts the orientation of the protein such that *trans*-pairing (dimerization across apposing membranes) is favored over *cis*-pairing (dimerization on the same membrane). This *trans*-pairing most likely functions as the “glue” for holding adjacent cisternae together^18^.

Interestingly, small interfering RNA (siRNA)-mediated depletion of either GRASP55 or GRASP65, or both, did not affect Golgi morphology at the light level, although EM and fluorescence recovery after photobleaching (FRAP) experiments revealed lateral unlinking of the Golgi^16, 19–22^. Light-induced inactivation (KillerRed) of GRASP55 or GRASP65 and GRASP65 knock-out mice again resulted in Golgi unlinking^23, 24^. While mitotic Golgi membranes are naturally unstacked and vesiculated during mitosis, they are inhibited for traffic, hence they cannot be used to study structure–function relationship of the Golgi^25, 26^.

We have recently demonstrated a new method to separate Golgi cisternae by re-targeting GRASP55 from the Golgi to the mitochondria upon addition of a dimerizing drug^19^. This resulted in the intercalation of mitochondria between Golgi cisternae so that they assume a “yeast-like” morphology where Golgi cisternae are unstacked.

Here, we have carefully characterized the artificially unstacked Golgi cisternae, which we call “land-locked cisternae” as they are “locked”, or immobilized on mitochondria. Despite the complete separation of *cis*-cisternae and *trans*-cisternae, coatomer, coated buds and vesicles were readily found on land-locked cisternae. Furthermore, elements of the vesicle budding and fusion machinery such as Arf1 and Rab1 cycled dynamically on and off the membranes of land-locked cisternae. We demonstrate that a wave of traffic through the Golgi or secretion out of the cell occurred in cells with land-locked cisternae, although the rate of transport was diminished compared to cells with stacked cisternae. Only intra-Golgi traffic was slowed in this system, most likely due to the increased intercisternal separation and the uncoupling of tether-mediated vesicular (anterograde, retrograde, or both) traffic. Finally, we monitored the *cis-*Golgi and *trans-*Golgi of land-locked cisternae in live cells by three-dimensional (3D) fluorescence microscopy. We found that cisternal identity is unchanged for at least 15 min, a time frame over which half-maximal transit of anterograde cargo through land-locked Golgi is accomplished. Thus, our data lend the first live cell imaging evidence in support of the stable compartments model and implicates COPI vesicles to mediate intra-Golgi traffic.

## Results

### Re-targeting GRASP55 redistributes the Golgi to mitochondria

**Fig. 1:**
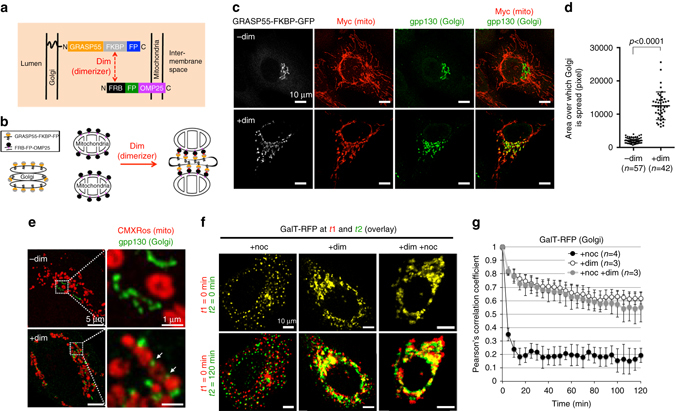
Re-targeting GRASP55 from Golgi to mitochondria generates “land-locked” Golgi cisternae. **a** Schematic of GRASP55 re-targeting assay, adapted from Supplementary Fig. 8a ^19^. GRASP55 is localized to the cytoplasmic face of the Golgi by N-terminal myristoylation. The C-terminal end of GRASP55 was fused to FKBP and a FP. OMP25 is anchored to the cytoplasmic face of the outer mitochondrial membrane by a C-terminal transmembrane domain. FRB and FP were fused to the N-terminal end of OMP25. Addition of the dimerizer induces FKBP and FRB heterodimerization. **b** Cartoon showing expected outcome of GRASP55 re-targeting to mitochondria. Addition of dimerizer induces heterodimerization of GRASP55-FKBP-BFP and FRB-FP-OMP25 leading to the adhesion of mitochondria to individual Golgi cisterna. **c** Confocal images of HeLa cells transfected with GRASP55-FKBP-GFP and FRB-Myc-OMP25 and immunostained for Myc and gpp130. Where indicated, cells were treated with dimerizer (2 μM) for 3 h. *Scale bar* is 10 μm. **d** Quantification of Golgi areas (gpp130 fluorescence) with and without dimerizer from **c**. **e** STED microscopy images of thin section (70 nm) HeLa cells that were transfected with GRASP55-FKBP-HA and FRB-Myc-OMP25 and stained for mitochondria and immunostained for gpp130. Where indicated, cells were treated with dimerizer (2 μM) for 3 h. *Scale bar* is 500 nm. **f** Live confocal imaging of Golgi in HeLa cells co-transfected with GalT-RFP, GRASP55-FKBP-BFP and FRB-GFP-OMP25, and pretreated with nocodazole, or dimerizer, or both. Overlay of frames from time 0 to time 0 and time 0 to time 120 min are shown. *Scale bar* is 10 μm. **g** PCC over time *t* of overlay images of GalT-RFP at time 0 to time *t* based on live image movies from **f**. *Error bars* = s.d. For statistical analysis, *t*-tests were used

The strategy for re-targeting GRASP55 was previously described^19^. Briefly, the C-terminus of GRASP55 was fused to an FKBP domain and a fluorescent protein (FP) to form GRASP55-FKBP-FP, leaving the N-terminus available for myristoylation and localization to the Golgi membrane^27^ (Fig. 1a). Then, we made the counterpart by fusing the N-terminus of OMP25, a mitochondria outer membrane protein^28^, to an FRB domain and a FP to form FRB-FP-OMP25 (Fig. 1a). Adding the dimerizer (A/C heterodimerizer, Clontech) results in the rapid tethering of GRASP55 to OMP25 on the mitochondria, leading to the ectopic adhesion of Golgi cisterna to mitochondria (Fig. 1b).

GRASP55 re-targeting was initiated after the cells were depleted of endogenous GRASP55 by siRNA-mediated silencing. Western blot analysis showed efficient replacement of endogenous GRASP55 by the siRNA-resistant form of GRASP55-FKBP-BFP (Supplementary Fig. 1a). Both GRASP55-FKBP-BFP and FRB-Myc-OMP25 localized correctly to the Golgi and mitochondrial outer membrane, respectively, as determined by immunofluorescence (Supplementary Fig. 1b, c). The stacking capability of GRASP55-FKBP-BFP was confirmed by its overexpression in GRASP55 RNA interference (RNAi)-mediated knockdown cells, where we observed the rescue of swollen Golgi back to flattened cisternae^19^ (Supplementary Fig. 1d). From hereon, we always silenced the endogenous GRASP55 by RNAi for 72 h before transfecting with an RNAi-resistant GRASP55-FKBP-FP and FRB-X-OMP25 for 18 h prior to the dimerizer-induced retargeting of GRASP55 to the mitochondria.

Next, we looked at Golgi architecture by immunofluorescence after re-targeting of GRASP55 (Fig. 1c). The normally perinuclear organization of the Golgi ribbon (labeled with *cis-*Golgi marker gpp130) was completely disrupted with the addition of dimerizer such that the Golgi was now spread out and colocalizing with the mitochondria network. Quantification revealed that the Golgi area increased by 6-fold, from an average of 2082 ± 822 pixels to 12,521 ± 4180 pixels in dimerizer-treated cells (Fig. 1d). Next, we looked at the redistribution of Golgi and mitochondria in GRASP55 re-targeted cells in real time by live imaging. We found that GRASP55-FKBP-BFP migrated to the mitochondria in 1 h, followed by the *trans-*Golgi (labeled with GalT-RFP) in 3 h (Supplementary Fig. 1e, f). This suggests that redirected GRASP55 was capable of pulling the rest of the Golgi membranes. We determined by immunofluorescence that both *cis-*Golgi and *trans-*Golgi markers migrated together, though the two never mixed even at the confocal level (Supplementary Fig. 1g). In addition, we showed that other *trans*-Golgi and *trans-*Golgi Network (TGN) markers (Furin, GalT, and TGN46) re-located to the mitochondria network after addition of the dimerizer (Supplementary Fig. 2). Importantly, the *trans*-Golgi and TGN markers co-localized with another *trans*-Golgi marker (p230), but remained distinct from *cis*-Golgi marker (gm130) (Supplementary Fig. 2), suggesting that the targeting of Golgi resident enzymes was not altered by Golgi membrane relocation.

In order to better visualize the Golgi-mitochondria structures, the same cells were also processed for thin-section STED (stimulated emission depletion) microscopy as previously described^29^. The cells were fixed and immunostained, then embedded in resin before they were cut into 70-nm thick sections. We observed a long chain comprising short Golgi pieces flanked by 500-nm long mitochondria only when dimerizer was present (Fig. 1e).

Given the dramatic reorganization of the Golgi in GRASP55 re-targeted cells, we wondered if overall intracellular organization would be disrupted as a whole. Interestingly, neither the ER network (PDI) or microtubule tracks (tubulin) were affected in GRASP55 re-targeted cells (Supplementary Fig. 3a, b). Interestingly, the ER exit site (ERES) (sec31) that is tightly associated to the Golgi^30^ also redistributed to the mitochondria, thereby maintaining its proximity to the Golgi (compare untransfected and transfected cells in Supplementary Fig. 3c).

Golgi membranes are transported unidirectionally to the microtubule organizing center (MTOC) by dynein, where they can form a ribbon by homotypic fusion^31^. We anticipated that the ectopic adhesion of Golgi to mitochondria in GRASP55 re-targeted cells would immobilize the Golgi as it adheres to the mitochondria. To test this, we compared the mobility of the induced, mitochondria-associated Golgi to the mobility of Golgi mini-stacks. Golgi mini-stacks are generated by depolymerizing microtubules with nocodazole treatment^32, 33^, hence they are slowly mobile via diffusion but no longer actively collect at the MTOC as does the Golgi ribbon. We imaged the Golgi every 5 min, then each time point was overlaid to time zero to calculate the degree of movement as a Pearson’s Correlation Coefficient (PCC) (Fig. 1f). The PCC of the mini-stacks with each other gradually declined from 1 to 0.2 (Fig. 1g), confirming that the mini-stacks are slowly mobile by diffusion. By contrast, those Golgi elements retargeted to mitochondria are largely stationary, as their PCC decreased much more gradually and plateaued at 0.6 (+dim; Fig. 1f, g). Furthermore, we monitored cells that were treated with both nocodazole and dimerizer (unstacked and retargeted to mitochondria). The PCC in these cells also declined very slowly to ~0.55, indicating that GRASP55 re-targeting successfully adhered previously mobile mini-stacks to the mitochondria as well (Fig. 1f, g).

We refer to these Golgi elements as “land-locked”.

### Re-targeting GRASP55 causes mitochondria to unstack Golgi

**Fig. 2:**
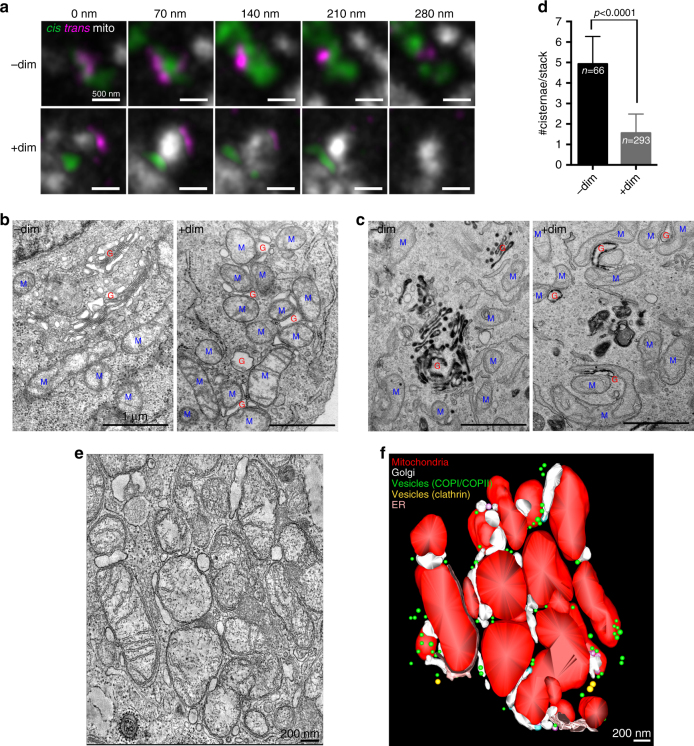
Land-locked Golgi cisternae are unstacked. **a** STED microscopy images of serial thin sections of HeLa cells cotransfected with GRASP55-FKBP-HA and FRB-Myc-OMP25 and stained for mitochondria (CMXRos) and immunostained for *cis*-Golgi (gpp130) and *trans-*Golgi (p230). Complete separation of *cis*-cisterna and *trans*-cisterna is evident only in the presence of the dimerizer. *Scale bar* is 500 nm. **b** TEM of HeLa cells transfected with GRASP55-FKBP-BFP and FRB-Myc-OMP25. *Blue* M indicates mitochondria. *Red* G indicates Golgi. *Scale bar* is 1 μm. **c** TEM of ManII-HRP HeLa cells transfected with GRASP55-FKBP-BFP and FRB-Myc-OMP25, then treated with DAB and H_2_O_2_ post-fixation to turn Golgi-specific membranes *black*. *Scale bar* is 1 μm. **d** Quantification of the number of cisternae/stack in GRASP55 re-targeted HeLa cells from **b**. **e** Optical section of an EM tomogram of HeLa cells transfected with GRASP55-FKBP-BFP and FRB-Myc-OMP25, then treated with dimerizer. *Scale bar* is 200 nm. **f** A surface-rendered 3D model of EM tomogram from **e** was generated to visualize the unstacked cisternae of land-locked Golgi. *Error bars* = s.d. For statistical analysis, *t*-tests were used

We prepared serial thin sections (70 nm) of GRASP55 re-targeted cells and visualized them by STED microcopy to test whether *cis* (gpp130) and *trans*-cisterna (p230) are unstacked in land-locked Golgi (Fig. 2a). Indeed, we were now readily able to identify unstacked Golgi, where the *cis*-cisternae and *trans*-cisternae were completely separated by mitochondria.

Next, we used transmission electron microscope (TEM) to characterize the extent of unstacking of our land-locked Golgi by mitochondria (Fig. 2b). Re-targeting GRASP55 reduced the number of cisternae from 4.9 ± 1.4 to 1.6 ± 0.9, indicating efficient unstacking of the Golgi (Fig. 2d).

While we surmised that the mitochondria-associated membranes in our EM images were derived from the Golgi, we decided to confirm this by using the ManII-HRP stable cell line^34^. These cells overexpress ManII-HRP, thereby serving as a general Golgi marker because the *medial-*Golgi enzyme is now distributed throughout the Golgi stack^34^. Treating these cells with 3,3′-diaminobenzidine (DAB) and H_2_O_2_ results in the specific deposition of DAB in Golgi cisternae due to expression of HRP in the Golgi lumen. When the cells are processed for EM, the Golgi appears as darkly stained membranes (Fig. 2c). Indeed, EM processing revealed heavy staining of the mitochondria-associated membranes, indicating that they were enriched in ManII-HRP. This demonstrates that the dimerizer-induced land-locked Golgi are specifically derived from the Golgi (Fig. 2c).

Given the extensive unstacking of Golgi produced by rerouting GRASP55, we tested the specificity of this phenomenon by rerouting other Golgi tethers (Supplementary Fig. 4). Rerouting the *cis-*Golgi stacking factor GRASP65 redistributed the Golgi to the mitochondria; however, the Golgi remained stacked (Supplementary Fig. 4a). Rerouting the *medial-*Golgi tether Golgin45 resulted in unstacking the Golgi as we observed for GRASP55 (Supplementary Fig. 4b, c). However, most of the green fluorescent protein (GFP)-Golgin45 was aggregated in the nucleus, which stems from its nuclear localization signal^35^; thus we disqualified this protein for our strategy as well. Rerouting the *trans-*Golgi tether Golgin97 did not result in redistribution of the Golgi to the mitochondria, but rather in the capture of transport vesicles (presumably COPI-coated) on mitochondria, consistent with previous reports^36^ (Supplementary Fig. 4d). Hence, we conclude that rerouting GRASP55 leads to the most efficient and targeted unstacking of Golgi.

We frequently observed cisternal swelling and shortening upon GRASP55 redistribution to the mitochondria in TEM images. However, given that the localization of Golgi enzymes is unaffected (Supplementary Fig. 2), it is likely that this morphological alteration is simply due to reduced adhesion of the FKBP-FRB-dimerizer interaction vs. the Golgin/GRASP interaction. In fact, we have previously reported swelling of Golgi cisternae upon silencing of GRASPs or Golgins by RNAi and determined this to be due to a decrease in adhesion energy^19^.

Finally, in order to gain a better picture of the extent of cisternal unstacking within land-locked Golgi, we performed double-tilt EM tomography on thick sections (~200 nm) of GRASP55 re-targeted cells (Fig. 2e). 3D reconstruction and modeling of the optical sections showed pervasive Golgi unstacking by mitochondria and an abundance of ~80 nm vesicles around the Golgi membranes (Fig. 2f).

### Land-locked Golgi contain coatomer, coated buds, and vesicles

**Fig. 3:**
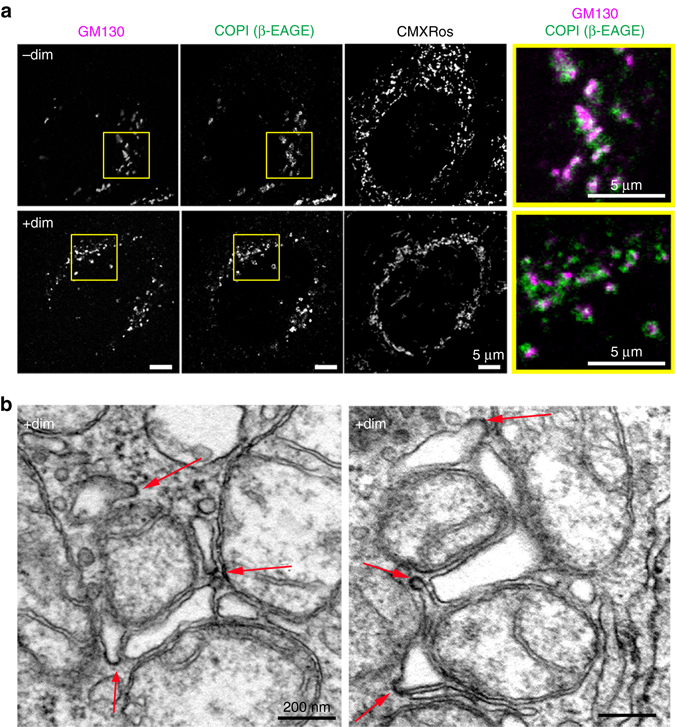
Land-locked cisternae contain coatomer, coated buds and vesicles. **a** Confocal images of HeLa cells transfected with GRASP55-FKBP-HA and FRB-Myc-OMP25, then stained for mitochondria and immunostained for GM130 and COPI. Where indicated, cells were treated with dimerizer (2 μM) for 3 h. *Scale bar* is 5 μm. **b** TEM images of vesicles and budded regions on land-locked Golgi in HeLa cells transfected with GRASP55-FKBP-BFP and FRB-Myc-OMP25 and treated with dimerizer. *Scale bar* is 200 nm

The coatomer COPI is a heptameric complex that is critical for intra-Golgi and *cis*-Golgi to ER traffic^37^. We immunostained against a COPI subunit (β-COP-specific anti-EAGE antibody) to test if COPI continues to be recruited to land-locked cisterna. We found that COPI formed patches on land-locked Golgi membranes as extensively as on normal Golgi membranes (Fig. 3a). By TEM, we saw numerous coated, budding regions on land-locked cisternae, as well as abundant ~80-nm sized vesicles characteristic of COPI (Fig. 3b and Supplementary Fig. 5a–i, Fig. 2d, e). We also found the ER lying very close to land-locked cisterna, with many vesicles lying in between. The ER membranes often harbored vesicle budding regions, indicative of ERESs (Supplementary Fig. 5j, k). This suggests that cargo can have direct access to land-locked Golgi after leaving the ER. Overall, the images from immunofluorescence, TEM, and tomographic EM data suggest that land-locked cisternae are engaged in active membrane traffic.

### Vesicle budding and fusion machinery on land-locked Golgi

The Golgi is a highly dynamic, membrane trafficking organelle that must receive virtually the entire output of the ER, sort, process, and then package these lipids and proteins into vesicles and transport carriers for targeting to their final destinations. We used FRAP to test if land-locked Golgi retained membrane fluidity and accessibility to regulators of membrane traffic while being adhered to mitochondria.

We first monitored GRASP55-FKBP-GFP by bleaching half of the Golgi, then checking for fluorescence recovery of GFP (Supplementary Fig. 6a, b). In normal Golgi (no dimerizer), GRASP55-FKBP-GFP recovered with a *t*_1/2_ of 18 ± 10 s and normalized fluorescence intensity of 42 ± 8% (Table 1). However, in land-locked Golgi (with dimerizer), GRASP55-FKBP-GFP recovered much more slowly with a *t*_1/2_ of 213 ± 40 s and normalized fluorescence intensity of 18 ± 5%. Thus, GRASP55-FKBP-GFP cycling between the Golgi and cytosol is strongly reduced when dimerizer is present, indicating that the “glue” between the Golgi and mitochondria is stable.

**Table 1:** Summary of FRAP analysis on normal or land-locked Golgi

	Normal (−dim)			Land-locked (+dim)		
	*t*_½max_ (s)	%max	*n*	*t*_½max_ (s)	%max	*n*
G55-FKBP-GFP	18 ± 10 s	42 ± 8%	3	213 ± 40 s	18 ± 5%	3
GFP-Rab1	49 ± 12 s	55 ± 5%	3	56 ± 18 s	47 ± 4%	4
Arf1-GFP	7 ± 3 s	28 ± 2%	3	8 ± 3 s	20 ± 5%	3

*Note*: *t*_½max_ refers to the time it takes to reach half the level of maximum fluorescence after photobleaching. %max refers to the % of normalized fluorescence recovered after photobleaching. Refer to Supplementary Fig. 6 for images

Next, we monitored two proteins of the Golgi trafficking machinery. GFP-Rab1 is a peripheral protein that plays an important role in modulating COPI recruitment to the *cis*-Golgi and in vesicle fusion^38–42^. In normal Golgi, GFP-Rab1 recovered with a *t*_1/2_ of 49 ± 12 s and normalized fluorescence intensity of 55 ± 5% (Table 1, Supplementary Fig. 6c, d). GFP-Rab1 recovered similarly on land-locked Golgi, with a *t*_1/2_ of 56 ± 18 s and normalized fluorescence intensity of 47 ± 4%. Next, we studied the dynamics of Arf1-GFP, a small GTPase that recruits the COPI coatomer complex to Golgi membranes to initiate vesicle budding^43^. In normal Golgi, Arf1-GFP recovered with a *t*_1/2_ of 7 ± 3 s and normalized fluorescence intensity of 28 ± 2% (Table 1, Supplementary Fig. 6e, f). Arf1-GFP recovered similarly on land-locked Golgi with a *t*_1/2_ of 8 ± 3 s and normalized fluorescence intensity of 20 ± 5%. The similar *t*_1/2_ and normalized fluorescence intensity values for both GFP-Rab1 and Arf1-GFP indicate that the trafficking machinery is cycling just as robustly on land-locked Golgi membranes as on normal Golgi membranes.

### Visualizing a wave of cargo through land-locked Golgi

**Fig. 4:**
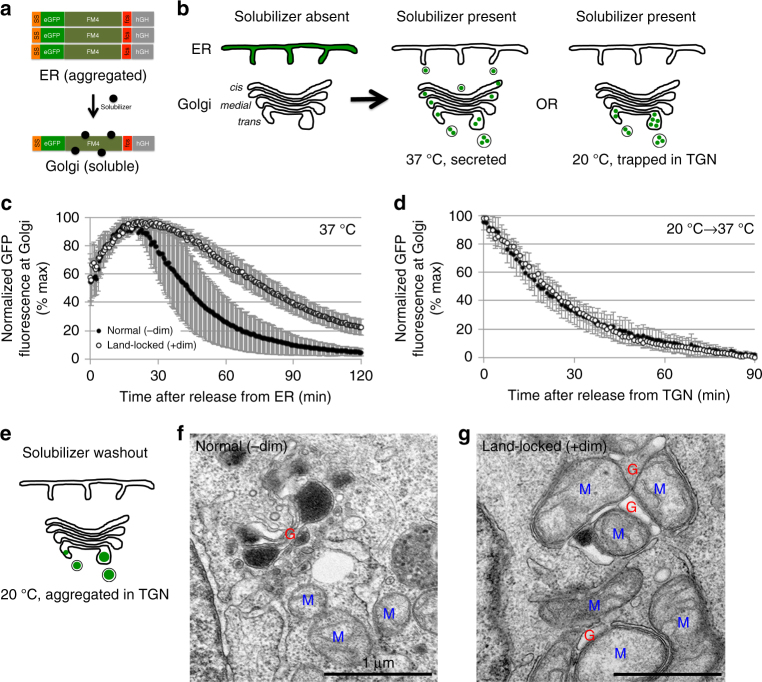
Live imaging a wave of anterograde cargo through individual normal or land-locked Golgi areas. **a** Cartoon schematic of the anterograde cargo ss-GFP-FM4-FCS-hGH. This cargo is aggregated and trapped in the ER until solubilizer is added. Then, the monomeric cargo is released from the ER for secretion. **b** Cartoon of ss-GFP-FM4-FCS-hGH cargo traffic and effect of temperature block. A wave of anterograde traffic is initiated from the ER by the addition of solubilizer at 37 °C, and the cargo is secreted out of the Golgi. However, at 20 °C, traffic is slowed so that the cargo accumulates at the TGN instead of getting secreted. **c** Quantification of a wave of cargo released at 37 °C from normal (*n* = 9) or land-locked (*n* = 9) Golgi in HT1080 cells that stably express ss-GFP-FM4-FCS-hGH (refer to Supplementary Fig. 6a, b for representative images and Table 2 for numbers). **d** Quantification of a wave of cargo after release from 20 °C block from normal (*n* = 9) or land-locked (*n* = 6) Golgi in HT1080 cells that stably express ss-GFP-FM4-FCS-hGH (refer to Supplementary Fig. 6c, d for representative images and Table 2 for numbers). The Golgi exit rate is almost indistinguishable between normal and land-locked Golgi. **e** Cartoon of the effect of 20 °C block combined with solubilizer washout on ss-GFP-FM4-FCS-hGH, which re-aggregates the cargo in the TGN. **f**, **g** TEM images of re-aggregated ss-GFP-FM4-FCS-hGH cargo in HT1080 cells that stably express ss-GFP-FM4-FCS-hGH. Large aggregated cargo can be seen in the lumen of **f** normal, stacked cisternae and **g** land-locked cisternae. *Scale bar* is 1 μm. *Error bars* = s.d

Given that land-locked Golgi are available to the trafficking machinery and positioned close to the ERES, we turned to live imaging to test whether land-locked Golgi can still accommodate and transport a wave of anterograde cargo. We used HT1080 fibrosarcoma cells that stably express a secretory cargo that aggregates reversibly in a drug-controlled fashion. The secretory cargo is composed of the signal sequence (ss), GFP, four repeats of the aggregation domain (FM4), furin cleavage site (FCS), followed by the human growth hormone (hGH)^44, 45^ (Fig. 4a). The ss-GFP-FM4-FCS-hGH protein is aggregated in the ER until the solubilizer (D/D solubilizing drug, Clontech) is added that causes its dissociation. The now monomeric protein can then exit the ER and enter the *cis*-face of the Golgi, undergo cleavage at the FCS in the *trans*-Golgi before secretion out of the cell.

Since we planned to image every 30 s per frame for 2 h, we controlled the experimental conditions to achieve no more than 10% photobleaching of GFP per 30 min (Supplementary Fig. 7a, b). We also only quantified movies where the cell did not drift out of the focal plane during the course of imaging, which was determined by simultaneously monitoring GalT-RFP fluorescence at the Golgi and only picking those cells whose red fluorescence remained constant throughout the experiment (Supplementary Fig. 7c, d). Controlling for photobleaching and drift within the experiment allowed us to monitor the change in GFP signal as a consequence of its secretion.

For live imaging of a wave of cargo, we induced the formation of land-locked Golgi by adding dimerizer for 3 h to transfected HT1080 cells. Then we added the solubilizer drug to dissolve the hGH cargo aggregates in the ER and immediately began imaging over a 2 h period (Supplementary Fig. 8a, b). Cycloheximide was present throughout the experiment to prevent new protein synthesis, and background fluorescence was subtracted for all of our quantification.

Quantification of cargo fluorescence (GFP) at the Golgi revealed that cargo entry into the Golgi (time until maximal intensity) was virtually the same for land-locked (24 ± 7 min) and normally stacked (20 ± 5 min) Golgi (Fig. 4c, Table 2). However, the rate of cargo exit from land-locked Golgi (half-time of 52 ± 7 min) was significantly reduced compared to normally stacked Golgi (half-time of 26 ± 13 min) (Fig. 4c, Table 2). We observed very similar Golgi entry and exit rates when we imaged every 5 min instead of every 30 s (Supplementary Fig. 9), confirming that photobleaching of GFP did not significantly contribute to the apparent rate of transport of GFP out of the Golgi.

**Table 2:** Summary quantification of a wave of cargo through individual normal or land-locked Golgi areas in living cells

Solubilizer added		Normal (−dim)	*n*	Land-locked (+dim)	*n*
37 °C	*t*_max_ (Golgi entry)	20 ± 5 min	9	24 ± 7 min	9
	*t*_½max_ (Golgi exit)	26 ± 13 min	9	52 ± 7 min	9
20 °C block	*t*_½max_ (Golgi exit)	19 ± 5 min	9	20 ± 4 min	6

*Note*: *t*_max_ (Golgi entry) refers to the time it takes for cargo to completely fill up the Golgi. *t*_½max_ (Golgi exit) refers to the time it takes for half of the cargo to exit the Golgi. 20 °C block refers to 20 °C TGN block followed by temperature shift to 37 °C at *t* = 0. Refer to Fig. 4 for images and chart

These results establish that ER to Golgi transport proceeds at normal speeds to the land-locked cisternae, which is not surprising based on our TEM images that show that the ERES remain close to land-locked cisternae (Supplementary Fig. 5j, k). The subsequent delay in exit from the Golgi could in theory result from either slower anterograde intra-Golgi transport or slower exit from the TGN, or a combination of both.

To distinguish between these two possibilities, we took advantage of the 20 °C temperature block that markedly stalls cargo exit from the TGN^46, 47^ (Fig. 4b). If the difference in trafficking observed between land-locked and unperturbed Golgi is *not* caused by different rates of exit from the TGN, the rates should be similar upon release of the 20 °C block. Indeed, half of the cargo exited from normally stacked Golgi in 19 ± 5 min after the temperature block was reversed as compared with 20 ± 4 min for the land-locked Golgi (Fig. 4d, Table 2), indicating that exit from the TGN is unimpaired in cells with land-locked Golgi.

In order to confirm that secretory cargo directly traffics through land-locked Golgi, we decided to use EM to visualize reaggregated ss-GFP-FM4-FCS-hGH cargo trapped in the Golgi using the 20 °C block (Fig. 4e). For this, we incubated the cells with solubilizer at 20 °C to allow cargo accumulation in the TGN, then washed away the solubilizer for 1 h at 20 °C to reaggregate the cargo before processing for EM. We observed big cargo aggregates in the lumen of normal Golgi cisterna as well as land-locked cisterna (Fig. 4f, g). The mitochondria-associated membrane is most likely a *trans-*Golgi cisterna given the lack of clathrin coats that typically decorate the TGN. The presence of cargo in the *trans-*Golgi during a TGN temperature block is not surprising, as we would expect the wave of cargo that is released to accumulate not only in the TGN but also back up to the *trans-*Golgi^48^. Overall, our results confirm that cargo are directly transiting land-locked Golgi as they do in normal Golgi.

### Bulk secretion through land-locked Golgi

**Fig. 5:**
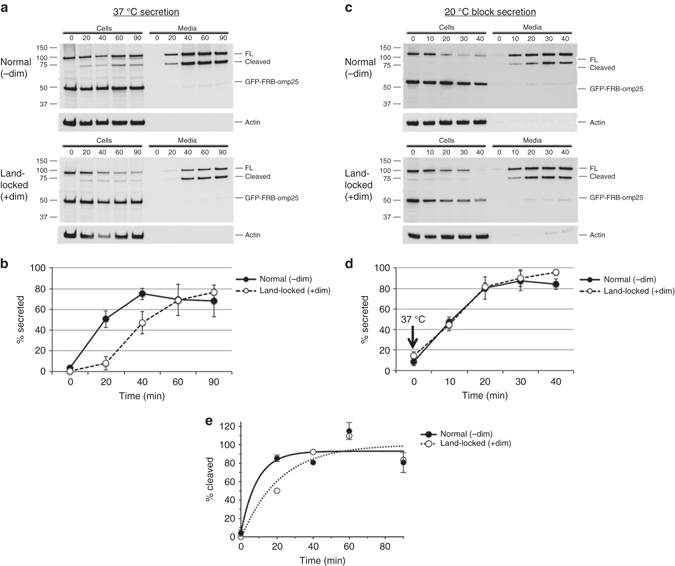
Bulk secretion through land-locked Golgi. **a** HT1080 cells that stably express ss-GFP-FM4-FCS-hGH were transfected with GRASP55-FKBP-HA and FRB-GFP-OMP25, then treated with dimerizer for 3 h before initiating secretion by adding solubilizer. Secretion was carried out at 37 °C in the presence of cycloheximide. For each time point, 10% of chase media and cell lysates were loaded for analysis by SDS-PAGE and Western blot with GFP and actin antibodies. Experiments were done in triplicate. **b** Quantification of Western blots from **a**. The % of cargo (full-length and cleaved) secreted was determined by taking the ratio of the secreted cargo (medium) over total cargo (medium + cell). **c** Secretion after release from 20 °C block. Cells were pre-incubated in dimerizer for 3 h at 37 °C, then cooled to 20 °C for 1 h before adding solubilizer for 2 h, then warmed back to 37 °C to initiate the secretion assay. For each time point, 10% of chase media and cell lysates were loaded for analysis by SDS-PAGE and Western blot with GFP and actin antibodies. Experiments were done in triplicate. **d** Quantification of Western blots from **c**. **e** Normalized furin cleavage efficiency of ss-GFP-FM4-FCS-hGH for normal and land-locked Golgi was calculated for each time point from cell fractions in **a** by taking the ratio of cleaved/(full-length + cleaved), and plotting that as a percentage of the maximum secreted amount. The data were fit to a single exponential curve and normalized to the plateau observed for furin cleavage in land-locked Golgi. *Error bars* = s.d

To confirm and extend the observations made on individual Golgi areas, we studied the kinetics of cargo processing and transport in the entire population of fibrosarcoma cells that stably express the ss-GFP-FM4-FCS-hGH cargo. Transfection of these cells with the GRASP55 re-targeting plasmids was about 80% efficient, with only a low level of expression variability from cell to cell. Similar to live imaging of a wave of cargo, we added dimerizer for 3 h to form land-locked Golgi. Then we added solubilizer to initiate traffic, and cycloheximide to prevent new protein synthesis, collecting the medium and cell lysate at time *t* post release for SDS-PAGE and Western blot analysis (Fig. 5a). Cycloheximide was present throughout the experiment to prevent new protein synthesis. For this type of assay, conclusions can only be drawn from the ratios of secreted cargo to total, i.e., (medium)/(medium + cells) × 100 (Fig. 5b). It took 40 min for the cargo to reach maximal secretion levels in the medium of normal Golgi cells, and 60 min in land-locked Golgi cells (Fig. 5a, b, Table 3). Next, we used the 20 °C block to determine post-Golgi traffic times. For both normal and land-locked Golgi cells, post-Golgi traffic took 20 min to reach maximal secretion levels (Fig. 5c, d, Table 3), consistent with our results from the study of single Golgi areas that showed that post-Golgi traffic is unaffected.

**Table 3:** Summary quantification of bulk secretion and processing through normal or land-locked Golgi cells

	Normal (−dim)	Land-locked (+dim)
*t*_max_ (37 °C)	40 min	60 min
*t*_max_ (20 °C)	20 min	20 min
*Cis* → *Trans*	20 min	40 min

*Note*: *t*_max_ (37 °C) refers to the time at which secretion begins to plateau when temperatures are held constant at 37 °C. *t*_max_ (20 °C) refers to the time at which secretion begins to plateau when temperatures are returned to 37 °C at *t* = 0 after a 20 °C block that accumulates cargo in the TGN. *Cis* → *Trans* refers to the time it takes for intra-Golgi traffic derived by subtracting *t*_max_ (20 °C) from *t*_max_ (37 °C). Refer to Fig. 5 for images and chart

Given that the ERES is juxtaposed to land-locked cisternae in our TEM images (Supplementary Fig. 5j, k), and Golgi entry time is almost identical in normal and land-locked Golgi cells in our live imaging traffic assay (Fig. 4c, Table 2), we made the assumption that ER to *cis-*Golgi time is fast as compared to later steps. Then, we can derive the intra-Golgi traffic time by subtracting the time it takes to reach maximum secretion after release from the 20 °C block from the time it takes to reach maximum secretion at 37 °C, i.e., *t*_max_ (37 °C)–*t*_max_ (20 °C). We thereby calculated a population average *cis-*to-*trans* transit time of 20 min for normal Golgi and 40 min for land-locked Golgi (Table 3), consistent with the data obtained with individual Golgi areas. The 20 min intra-Golgi traffic time of normal Golgi cells is within range of the 15 min reported for other cargo such as vesicular stomatitis virus G glycoprotein (VSV-G) and procollagen^49–51^.

We also quantified Golgi processing ability while monitoring the arrival of the cargo in the TGN by measuring furin cleavage efficiency of the hGH cargo in the cell fraction during secretion at 37 °C. We already know that the TGN localization of furin is unperturbed in land-locked Golgi cells (Supplementary Fig. 2a, b). At the first time point *t* = 20 min, the cargo was already 80% cleaved in normal Golgi cells and 50% cleaved in land-locked Golgi cells. By the next time point *t* = 40 min, land-locked Golgi cells have caught up with normal Golgi cells, and by *t* = 60 min maximum furin cleavage efficiency is attained (Fig. 5e). Thus, the processing efficiencies are the same but the rate is slower for land-locked Golgi cells. This can be attributed to the slower intra-Golgi traffic rate of land-locked Golgi cells. Most likely at *t* = 20 min, the bulk of the cargo is still stuck in the *cis* or *medial-*Golgi, unable to get cleaved by furin that is located in the TGN. But by the next time point of *t* = 40 min, the furin cleavage efficiency has already plateaued to maximum levels. When we fit our data (normalized to the plateau observed for furin cleavage in land-locked Golgi) to a single exponential curve, we determined the half-maximal time of *cis* to *trans* traffic in land-locked Golgi to be 15 min.

Altogether, our bulk secretion results demonstrate that processing can occur with normal efficiency and transport can occur in a timely manner (two-fold slower, from 20 min to 40 min). This delay is most likely due to slowed intra-Golgi traffic, since neither ER-Golgi traffic nor post-Golgi traffic is impaired in land-locked Golgi cells.

### Land-locked cisternae are stable and do not mature

With the increased spatial separation of cisternae in land-locked Golgi, as well as their demonstrated ability to traffic and process anterograde cargo, we examined mammalian Golgi dynamics by 3D time-lapse confocal microscopy. Compared to yeast Golgi cisternae, our land-locked Golgi cisternae have the advantage of being largely stationary (Fig. 1f, g), therefore facilitating their tracking. Since land-locked cisternae are effectively immobilized on mitochondria, cisternal *progression* during a wave of anterograde transport can be ruled out. However, cisternal *maturation*—a change in the biochemical composition of the cisternae unaccompanied by progression—may occur in land-locked Golgi, as land-locked cisternae could still receive retrograde COPI vesicles carrying resident enzymes that would allow them to mature from *cis* to *medial* to *trans* in situ.

To distinguish among these possibilities, we performed live imaging experiments by transiently transfecting HeLa cells with plasmids for GRASP65-GFP and GalT-RFP to demarcate *cis-*Golgi and *trans-*Golgi, respectively, together with the inducible land-locked Golgi plasmids (GRASP55-FKBP-BFP and FRB-Myc-Omp25). After 18 h transfection, we incubated the cells with nocodazole for 3 h prior to adding dimerizer for 3 h in order to facilitate unstacking of the Golgi^19^. Nocodazole prevents microtubule polymerization, hence we expected more extensive breakdown of the Golgi ribbon into mini-stacks without influencing secretion^32, 52–54^. We also know from earlier experiments that nocodazole does not affect the extent of immobilization of Golgi cisternae land-locked to mitochondria (Fig. 1f, g). Once the drug treatment was complete, we imaged the cells by capturing a *z*-stack of 20 optical sections (500-nm thick) every 1 min for 30 min.

**Fig. 6:**
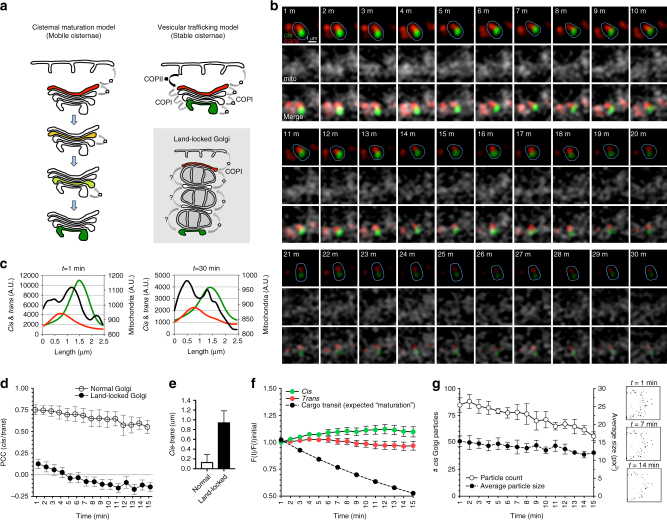
Land-locked cisternae are stable and do not mature. **a** Cartoons showing the two main intra-Golgi trafficking models and the land-locked Golgi. The cisternal maturation model postulates that the cisternae are mobile, maturing from *cis* to *medial* to *trans* as they progress through the stack. These mobile cisternae serve as anterograde cargo carriers, processing the cargo within as they uptake resident enzymes from older cisterna by retrograde COPI vesicles. The stable compartments model postulates that the Golgi exists as a static compartment that receives and exports cargo through anterograde COPI-coated vesicles. Cisternal identity is preserved by the recycling of any leaked enzymes in retrograde COPI vesicles. In land-locked Golgi, the cisterna are separated and adhered to mitochondria, increasing the spatial separation between cisternae from ~30 to ~500 nm. While a role for recycling Golgi enzymes by retrograde COPI vesicles is established, the question mark denotes how it is unclear what mediates anterograde traffic. **b** Live imaging land-locked Golgi. HeLa cells were transfected with GRASP55-FKBP-BFP and FRB-Myc-Omp25 to be able to re-target Golgi to the mitochondria, as well as GRASP65-GFP to mark the *cis-*Golgi and GalT-RFP to mark the *trans-*Golgi. Cells were treated with nocodazole to break the Golgi ribbon into mini-stacks, and then with dimerizer to produce land-locked Golgi. Then the cells were imaged every minute as a z-stack for 30 min. A single confocal section of a land-locked Golgi whose *cis-*Golgi and *trans-*Golgi markers flank a mitochondria is followed over time (encircled in *blue*). **c** Plot profiles were taken across land-locked Golgi shown in **b** for *cis-*Golgi (GRASP65-GFP, *green*), *trans-*Golgi (GalT-RFP, *red line*), and mitochondria (GRASP55-FKBP-BFP, *black line*) fluorescence. *Cis* and *trans-*Golgi markers remain well separated at *t* = 1 min and *t* = 30 min. **d** PCC was calculated for GRASP65-GFP and GalT-RFP for normal Golgi (*n* = 7) and land-locked Golgi (*n* = 20) over the course of 15 min on merged planes. *Error bars* = s.e.m. **e** Quantification of the mean distance between the peak fluorescence of *cis* and *trans-*Golgi markers for normal Golgi (*n* = 7) and land-locked Golgi (*n* = 20). *Error bars* = s.d. **f** Quantification of the integrated intensity of *cis* and *trans*-Golgi markers for land-locked Golgi over time (merged planes, *n* = 20). The inversed rate of hGH arrival at the *trans*-Golgi (Fig. 5e) is plotted for comparison (expected rate of maturation). *Error bars* = s.e.m. **g** Quantification of the average number (left *y*-axis, *triangles*) and area (right *y*-axis, *circles*) of *cis*-Golgi particles on merged planes from different cells and experiments (*n* = 3). *Inset*: visual correlates for thresholded *cis* Golgi-marker positive particles tracked at *t* = 1, 7, and 14 min. *Error bars* = s.e.m

If cisternal maturation occurred, then we would expect to see the green *cis-*Golgi cisternae maturing into a red *trans-*Golgi cisternae, i.e., losing the *cis-*Golgi stacking factor GRASP65-GFP and acquiring the *trans*-Golgi enzyme GalT-RFP (Fig. 6a). However, if the cisternae remain stable, then both *cis* and *trans-*Golgi markers will remain green and red, respectively.

Figure 6b shows an example of land-locked *cis-*Golgi (GRASP65-GFP) and *trans-*Golgi cisternae (GalT-RFP) flanking a mitochondria over the course of 30 min (more examples are provided in Supplementary Fig. 10), and their immobilization onto mitochondria was supported by the fact that they only moved 2–3 confocal planes throughout the movie.

Contrary to yeast Golgi where cisternal maturation was readily observed^12, 13^, and where mitochondria-association of Golgi membranes resulted in the mixing of early and late compartments^55^, we discovered that our land-locked *cis*-Golgi and *trans*-Golgi compartments were stable over the course of 30 min (Fig. 6b, c and Supplementary Fig. 10). In land-locked Golgi, the half-maximal rate of cargo arrival at the *trans*-Golgi was determined to be 15 min (Fig. 5e). According to the model of cisternal maturation, this rate should be reflected by a concomitant change in the biochemical composition of the cisternae (*cis* to *trans*), as it was observed in yeast^12, 13^. Therefore, we tracked 20 examples of land-locked *cis*-Golgi and *trans*-Golgi compartments from different cells and independent experiments for 15 min (Supplementary Fig. 11).

We tested for content mixing (conversion) of the subcompartments by monitoring the PCC for merged planes of each time point (Fig. 6d). Throughout a 15-min period, at which significant content mixing should have been observed had maturation occurred, we did not observe any colocalization of our markers (PCC <0.1; *n* = 20). Supportively, the distance between the *cis-*Golgi and *trans-*Golgi averaged 0.94 μm (Fig. 6e), which corresponds to the size of a mitochondria. On the contrary, in our control cells that were only treated with nocodazole but no dimerizer (Supplementary Fig. 12), the PCC for the *cis-*Golgi and *trans-*Golgi markers stayed high around 0.75 (*n* = 7, Fig. 6d). The high Pearson’s value is most likely due to overexpression of the Golgi markers, as we observed very low or negative Pearson’s values when immunostaining for endogenous Golgi markers^56^. The distance between the *cis* and *trans-*Golgi markers averaged 0.13 μm (*n* = 7, Fig. 6e).

Next, we quantified the integrated intensity of GalT-RFP and GRASP65-GFP for the 20 examples of landlocked *cis* and *trans*-Golgi cisternae for 15 min (Fig. 6f, Supplementary Fig. 11), and plotted the inversed rate of cargo arrival at the *trans*-Golgi from our bulk secretion assay (Fig. 5e) as the expected rate of maturation (*trans*-Golgi localization of Furin was established in Supplementary Fig. 2a, b). As the cisternal maturation model predicts, the *cis*-Golgi cisternae should convert into later cisternae by acquiring their composition over time, and hence we would have expected a significant reduction in the intensity of either marker over time should maturation occur. In fact, for the 20 pairs of land-locked *cis* and *trans*-cisternae that we tracked, neither of the markers ever deviated more than 10% off the initial measured intensity (Fig. 6f, Supplementary Fig. 11). Another prediction from the maturation model would be the appearance of *cis*-Golgi marker-positive compartments in the cells over time (newly formed cisternae). We tracked several areas of cells for any change in the amount of *cis* Golgi-positive objects, and monitored their average area (Fig. 6g). Again, both the average number of particles observed as well as their relative area remained largely unaffected.

In summary, in our system where the Golgi is unstacked and “land-locked” to mitochondria, we observe that (i) trafficking of cargo still occurs and is merely slowed down 2-fold, (ii) the immobilized *cis* and *trans*-Golgi cisternae are stable and do not exhibit conversions, and (iii) their composition, size, and area do not change at a rate that would be required to explain the rate of cargo trafficking in this system.

We therefore are prompted to conclude that, unlike yeast Golgi, individual mammalian Golgi cisternae represent stable entities with a specific set of enzymes. Anterograde transport of cargo is then achieved by carriers/machinery that allow for a vectorial transport, while leaving a stable pool of enzymes behind.

## Discussion

Re-targeting of the stacking protein GRASP55 effectively unstacks the Golgi by introducing an ectopic adhesion between mitochondria and Golgi cisternae to produce immobilized “land-locked” cisternae. *Cis-*Golgi and *trans*-Golgi localization is retained even after this massive perturbation, indicating that the separated land-locked cisternae retain their identities. The acute, inducible redistribution of organelles probably contributed to the success of Golgi unstacking, since previous attempts that knocked down or knocked out stacking factors were long-term treatments that produced minor impairments like Golgi swelling, unlinking or reduced stacking^16, 19–24^. The land-locked cisternae are immobilized once they are adhered to the mitochondria, *cis*-Golgi and *trans*-Golgi cisterna are efficiently unstacked by mitochondria, and land-locked cisternae are accessible to the trafficking machinery and can directly mediate anterograde traffic.

We were surprised that re-targeting GRASP55, a *medial-*Golgi protein^14^, can pull portions of the entire Golgi stack to the mitochondria, given that GRASP55 is a peripheral protein that associates with the Golgi through an N-terminal myristoyl group and a central Golgin45 binding domain^27, 57^. The tight association to the Golgi was also unexpected because GRASP55 rapidly cycles on and off the Golgi membrane as determined by our FRAP analysis. Our result vividly confirms a potent and specific role for GRASP55 in Golgi stacking, especially since re-targeting the *cis-*Golgi stacking factor GRASP65 did not unstack the Golgi even though it did bring the Golgi stack to the mitochondria. Furthermore, consistent with earlier studies^36^, other Golgi proteins such as Golgin84, Golgin97, GCC88, TMF captured transport vesicles but did not capture cisternae, consistent with their proposed functions as Golgi tethers.

It is amazing that cargo processing and transport continue with normal efficiency and are merely slowed down when the normal topological relationships among Golgi cisternae are grossly altered and the individual cisternae are frequently fully separated from each other and held at great distance and moreover trapped within a glued matrix of adherent mitochondria. This is all the more remarkable given that cisternal stacking is such a highly conserved feature of the Golgi.

The simplest explanation of delayed transport among land-locked cisternae would be the greater role now played by diffusion. When the cisternae are closely apposed, vesicles can be essentially handed off directly from one cisternae to another, only rarely dissociating from the stack or the matrix of tethers surrounding it. When the cisternae are separated by more than the molecular reach of the tethers, now the vesicles must diffuse to reach their destination. Instead of traveling merely 10–20 nm (the normal separation of adjacent cisternae in a stack), the vesicle must now travel ~0.5 μm or more because of the inter-leaved mitochondria. Possibly this diffusion process would be faster, were it unconstrained in three dimensions, because in land-locked Golgi areas the vesicles are limited to exploring the narrow channels of space among the adherent mitochondria (see TEM in Fig. 2b and EM tomo in Fig. 2e). A second reason maybe the inhibition of tubular connections that have been observed between Golgi cisternae and are proposed to form a “fast-track” for secretory cargo. The cisternal progenitor model proposes that these tubular connections are formed by homotypic fusion of matching Rab domains on neighboring cisternae^58^. Anterograde traffic through such tubular connections will become highly unlikely in land-locked (unstacked) Golgi given that *cis* and *trans-*Golgi are on average ~1 μm apart.

Previous studies that manipulated GRASP proteins to disrupt Golgi morphology reported modestly accelerated traffic rates. GRASP55/GRASP65 knock down led to enhanced traffic of the VSV-G, the cell adhesion protein integrin, and the lysosomal enzyme cathepsin D^22^, as well as CD8^19^. Microinjection of GRASP65 antibody resulted in a 2-fold increase in CD8 traffic^59^. The authors suggest that reduced stacking increases the Golgi surface area that is available to bind coat proteins for vesicle budding and fusion, thereby increasing the rate of protein transport. In support of this, the rate and efficiency of COPI vesicle formation in vitro was higher for unstacked Golgi than stacked Golgi^59^, and COPI subunits became more readily membrane bound in cells depleted of GRASP55 and GRASP65^22^. In these studies the cisternae were partially but not completely de-adhered and remain close; in our studies they are physically dissociated. This is a key difference, as when the cisternae remain proximate the vesicles can still be directly handed off, whereas when they are distant (our case) they must diffuse. This would mask the small acceleration effect noted previously in land-locked Golgi.

Given the slower flux out but normal flux in, cargo will concentrate in land-locked cisternae. In a stacked Golgi, increased cargo load leads to cisternal swelling at the rims, where transport takes place^45, 60^ (Fig. 4f), as the central region of the cisterna are flattened by the strong adhesive interaction of GRASPs and Golgins. Without these adhesive forces, land-locked cisternae can freely expand with increased cargo load, and this may be another contributing factor to the swollen cisternae phenotype we observed in our EM images.

Live imaging our land-locked mammalian Golgi system has provided new evidence for the stable compartments model for the stacked Golgi in animal cells. We failed to observe maturation of cisternae during cargo transport, using essentially the same criteria that readily demonstrated maturation of the naturally dissociated cisternae of budding yeast^12, 13^. The fact that different secretory cargoes can traverse the same Golgi at different rates (e.g., 2 min for albumin, 15 min for VSV-G and procollagen^49–51^), and that Golgi exit is a non-linear process^8^ suggests that the stable compartments model can readily accommodate these phenomena, as well as explain the existence of biochemically separate compartments^61, 62^, the gradient of glycosylation enzymes^6, 63–66^, and the occurrence of secretory cargo waves. Our observations demonstrate that dissociative transport between stable cisternae can take place in the absence of maturation, and that this is sufficient for efficient secretion. They do not rule out that cisternal maturation may occur in intact stacks, of course. However, our results suggest that it is unlikely to be the major contributor to the kinetics of cargo transport in the stacked Golgi of animal cells.

## Methods

### Plasmids

The cloning of GRASP55-FKBP-FP and FRB-Myc-OMP25 into pC4 were previously described^19^. GRASP65 and Golgin97 were similarly cloned after digesting the pC4 vector with Xba1 for their insertion into the 5′ end of the FKBP-FP tag. GFP-Golgin45 was inserted into the SpeI site of the pC4 vector that fuses it to the 3′ end of FKBP. The cloning of ssGFP-FM4-FCS-hGH was previously described^44^. GFP-Rab1 was synthesized in our lab by Intaek Lee. GalT-RFP was a gift from Derek Toomre (Yale University, USA).

### Cell culture

HeLa cells (ATCC CCL-2) were grown at 37 °C in 5% CO_2_ in DMEM (Gibco, Grand Island, NY, USA) supplemented with 10% FBS (Gibco). The HeLa ManII-HRP stable cell line was a gift from Franck Perez^34^. The HT1080 human fibrosarcoma stable cell line expressing ssGFP-FM4-FCS-hGH was previously generated in our lab by Allen Volchuk^45^. Both HeLa ManII-HRP stable cell line and the HT1080 stable cell lines were grown under the same culture conditions as HeLa cells, except with the addition of 0.5 mg/ml geneticin and 50 U/ml penicillin-streptomycin (Gibco) to their medium.

### Transfection

Plasmid transfections were performed with Lipofectamine 2000 (Invitrogen, Grand Island, NY, USA) as recommended by the manufacturer. siRNA transfections were performed with RNAiMax (Invitrogen) as recommended by the manufacturer. Human GRASP55 siRNAs were made by Integrated DNA Technology (Coralville, IA, USA) with the following target sequences: GRASP55 #1 (5′-CCACCAGGAACUACAGGAAUUGAAC-3′) and GRASP55 #2 (5′-CUGCGAGAGACCUCAGUCACACCAA-3′).

### Re-targeting GRASP55 assay

200,000 HeLa (or HeLa ManII-HRP stable) cells were seeded 1 day before siRNA treatment into a 6-well plate. After 24 h knockdown with GRASP55 #2 siRNA, the cells were trypsinized and split into four wells of a 6-well plate or 35 mm Glass Bottom Microwell Dishes (MatTek, Ashland, MA, USA). After 72 h knockdown, the cells were transfected with GRASP55(res)-FKBP-FP and FRB-FP-OMP25 for 18 h. FKBP-FRB heterodimerization was initiated by adding 2 μM A/C heterodimerizer (Clontech Laboratories, Mountain View, CA, USA) for the indicated amount of time at 37 °C.

### Live cell imaging a wave a cargo

200,000 HT1080 stable cells expressing the ssGFP-FM4-FCS-hGH cargo were seeded on 35 mm Glass Bottom Microwell Dishes (MatTek, Ashland, MA, USA) 1 day before plasmid transfection. 18 h post-transfection, cells were treated with 2 μM A/C heterodimerizer (Clontech Laboratories, Mountain View, CA, USA) in growth medium. Then we exchanged buffers to Hanks’ balanced salt solution (HBSS; Life Technologies) supplemented with 10% fetal bovine serum (Life Technologies) and 100 μg/ml cycloheximide (Sigma-Aldrich, St. Louis, MO) for imaging. We added 1 μM D/D Solubilizer (Clontech Laboratories, Mountain View, CA, USA) to initiate a wave of cargo from the ER. We used a Yokagawa-type spinning disc confocal microscope system for fast imaging (PerkinElmer). The system was mounted onto an inverted microscope (IX-71; Olympus) quipped with a 1 Kb × 1 Kb EM charge-coupled device camera (Hamamatsu Photonics). The spinning disc confocal microscopy (SDCM) system was controlled by Volocity (Perkin Elmer), cells were imaged by using a 63 × 1.4 NA oil objective lens, and exposure time was 200 ms.

### Live cell imaging land-locked Golgi

200,000 HeLa cells were seeded on 35 mm Glass Bottom Microwell Dishes (MatTek, Ashland, MA, USA) 1 day before plasmid transfection. 18 h post-transfection, cells were treated with 2 μg/ml nocodazole (Sigma-Aldrich, St. Louis, MO) for 3 h in growth medium, and then 2 μM A/C heterodimerizer (Clontech Laboratories, Mountain View, CA, USA) was added for another 3 h. Then we exchanged buffers to HBSS (Life Technologies) supplemented with 10% fetal bovine serum (Life Technologies) and 100 μg/ml cycloheximide (Sigma-Aldrich, St. Louis, MO) for imaging. We used a Yokagawa-type spinning disc confocal microscope system for fast 4D imaging (PerkinElmer). The system was mounted onto an inverted microscope (IX-71; Olympus) quipped with a 1 Kb × 1 Kb EM charge-coupled device camera (Hamamatsu Photonics). The SDCM system was controlled by Volocity (Perkin Elmer), cells were imaged by using a 63 × 1.4 NA oil objective lens, and exposure time was 200 ms. PCC was determined by generating brightest-point Z-projections of each time series, thresholding the images, and employing the ImageJ-plugin “Colocalization Test”, for each time point and with the cis Golgi signal as a region of interest (ROI). On the raw data, the integrated intensity of the respective cisternal markers were quantified over time using the ImageJ-plugin “Time Series Analyzer V3”. The number and area of particles over time for images containing thresholded landlocked *cis*-Golgi cisternae was quantified using the Image-plugin “Analyze Particles”.

### Secretion assay

300,000 HT1080 stable cells expressing the ssGFP-FM4-FCS-hGH cargo were grown on 6-well plates. Secretion assays were performed in HBSS (Life Technologies) supplemented with 1% fetal bovine serum (Life Technologies) and 100 μg/ml cycloheximide (Sigma-Aldrich, St. Louis, MO). Incubations at 20 and 37 °C were performed in temperature-controlled incubators. Traffic was initiated by adding 1 μM D/D solubilizer (Clontech, Mountain View, CA), then at the indicated time points, chase media were collected and precipitated with 10% v/v trichloroacetic acid (Sigma-Aldrich, St. Louis, MO), washed in acetone, then dried. Cells were scraped and pelleted. Chase medium and cell extracts were separated by SDS-PAGE, then transferred onto nitrocellulose membrane for immunoblotting. After blocking with 5% w/v fat-free milk in PBS-T, the membranes were incubated with the appropriate primary antibodies. We used anti-Tag(CGY)FP (Evrogen, Moscow, Russia), anti-actin (Cell Signaling Technology, Danvers, MA), anti-GRASP55 (Proteintech, Rosemont, IL), anti-GAPDH (Sigma-Aldrich, St. Louis, MO). Then we used appropriate secondary antibodies conjugated to horse-radish peroxidase (Santa Cruz Biotechnology, Santa Cruz, TX) for chemiluminescence detection.

### Immunofluorescence and Confocal/STED microscopy

Cells were seeded on glass coverslips in 24-well plates. When indicated, cells were stained with Red Mitotracker CMXRos (Molecular Probes) at 500 nM in Live Cell Imaging Solution (Molecular Probes) for 30 min at 37 °C. Cells were fixed in 4% paraformaldehyde in PBS for 20 min at RT. Cells were then permeabilized in permeabilization buffer (0.3% NP-40, 0.05% Triton X-100, 0.1% BSA (IgG free), 1× PBS) for 3 min at RT, blocked in block buffer (0.05% NP-40, 0.05% Triton X-100, 5% goat serum, 1× PBS) for 1 h, incubated in 1:1000 primary antibody in block buffer at 4 °C overnight, washed twice in wash buffer (0.05% NP-40, 0.05% Triton X-100, 0.2% BSA (IgG free), 1× PBS), incubated with 1:1000 secondary antibody in block buffer for 1 h at RT, washed twice in PBS, then mounted on 1-mm thick precleaned microscope slides (Thermo Fisher Scientific, Waltham, MA) with ProLong Gold antifade reagent (Life Technologies, Carlsbad, CA). We used the following primary antibodies: anti-GM130 (1:1000, BD Biosciences, San Jose, CA), anti-gpp130 (1:1000, Covance, Princeton, NJ, Cat. No. 610822), anti-p230 (1:1000, BD Biosciences, San Jose, CA, Cat. No. 611280), anti-HRP (1:500, kind gift from Franck Perez, Institut Curie, France), β-EAGE (1:1000, kind gift from Thomas E. Kreis, EMBL, Germany), and Myc 9E10 purified mouse antibody (1:1000, kind gift from J. Michael Bishop, UC, San Francisco). All secondary antibodies conjugated to fluorescent dyes were purchased from Molecular Probes, except secondary antibodies conjugated to the STED dye Atto647N, which were purchased from Active motif. Cells were imaged using Zeiss LSM510 microscope with a 63× oil objective in the multi-tracking mode. Images were analyzed using the Zeiss LSM510 software or ImageJ.

For (serial) thin sectioning, samples were immunostained as described above, then post-fixed in 3% paraformaldehyde + 0.1% glutaraldehyde for 30 min. After dehydration with 100% Ethanol, the samples were infiltrated and embedded with UltraBed Low Viscosity Epoxy (EM Science) and cured for 48 h at 60 °C. 70–100 nm serial thin sections were cut using a Leica microtome and DiATOME Ultra 45 Diamond Knife. Dried sections were mounted onto 1.5 coverglass in mowiol. Thin sections were imaged using a commercial Leica TCS STED microscope with a 100× oil objective, with a pulsed diode laser (PDL 800-B, PicoQuant) emitting at 640 nm and a Ti-Sapphire laser (Mai Tai, Spectra Physics) for depletion. ATTO647N-labeled samples were depleted at 770 nm. Serial images were manually aligned in Adobe Photoshop, using the mitochondrial marker channel for rotational and translational alignment. Images were smoothed using a Gaussian blur of 1.5 pixels. 3D volumes were reconstructed in Volocity (Perkin Elmer).

### Electron microscopy

For regular Epon embedding, the cells were fixed in 2.5% gluteraldehyde in 0.1 M sodium cacodylate buffer pH 7.4 for 1 h at RT. After three rinses in 0.1 M sodium cacodylate, the cells were scraped in 1% gelatin and pelleted in 2% agar. Chilled blocks were trimmed and then post-fixed in 1% osmium tetroxide for 1 h, en bloc stained in 2% aqueous uranyl acetate in maleate buffer pH 5.2 for another hour, then rinsed and dehydrated in an ethanol series followed by resin infiltration (Embed 812, EM Science) and baked over night at 60 °C. Hardened blocked were cut using a Leica UltraCut UC7, 60 nm sections (or 250 nm sections for tomography) were collected onto formvar/carbon-coated nickel grids and contrast stained using 2% uranyl acetate and lead citrate.

For DAB labeling, cells were fixed in 1.2% gluteraldehyde in 0.1 M sodium cacodylate buffer pH 7.4 for 1 h at RT. After three rinses in 0.1 M sodium cacodylate, the cells were incubated in 0.1 M ammonium phosphate pH 7.4 for 10 min, followed by 10 min in 0.2 □m filtered 0.1 M ammonium phosphate pH 7.4 containing 1 mg/ml DAB for 10 min. Then, the cells were incubated in 0.1 M ammonium phosphate pH 7.4 containing 0.5 mg/ml DAB and 0.05% H_2_O_2_ for 10 min before washing three times in cold water. The cells were then fixed in 1% OsO_4_, 1% K_4_Fe(CN)_6_ in 0.1 M sodium cacodylate for 1 h at RT, washed three times in 0.1 M sodium cacodylate, washed three times in water, then stained overnight with 0.2 μm filtered 0.5% uranyl magnesium acetate. After washing three times in water, the cells were dehydrated in an ethanol series, infiltrated with a 1:1 mix of Epon and ethanol for 1 h, then with Epon for 2 h and baked 48 h at 65 °C. Thin sections of the DAB stained cells were cut the same as the unstained cells described above.

The 60-nm sections were viewed using a FEI Tecnai Biotwin TEM at 80 kV. Images were typically taken at 26,000× magnification with a Morada CCD and iTEM (Olympus) software.

The 250-nm sections for tomography were viewed using a FEI Tecnai TF20 at 200 kV with tilt angles from 60° to −60°. Data were collected using a FEI Eagle 4 K × 4 K digital camera. The volume reconstruction was done using IMOD and segmentation done using 3Dmod (Boulder Laboratory for 3D Electron Microscopy of Cells, University of Colorado).

### Data availability

The data that support the findings of this study are available from the authors on reasonable request, see author contributions for specific data sets.

## Electronic supplementary material

Supplementary Information
