# Supplementary material for: Land-locked mammalian Golgi reveals cargo transport between stable cisternae
Source: Nat Commun. 2017 Sep 5;8:432. doi: 10.1038/s41467-017-00570-z (PMC5585379; doi:10.1038/s41467-017-00570-z)
Supplement: Supplementary file 1 — Supplementary Information [file 41467_2017_570_MOESM1_ESM.pdf]

Description of Supplementary Files

Title: Supplementary Information

Description: Supplementary Figures

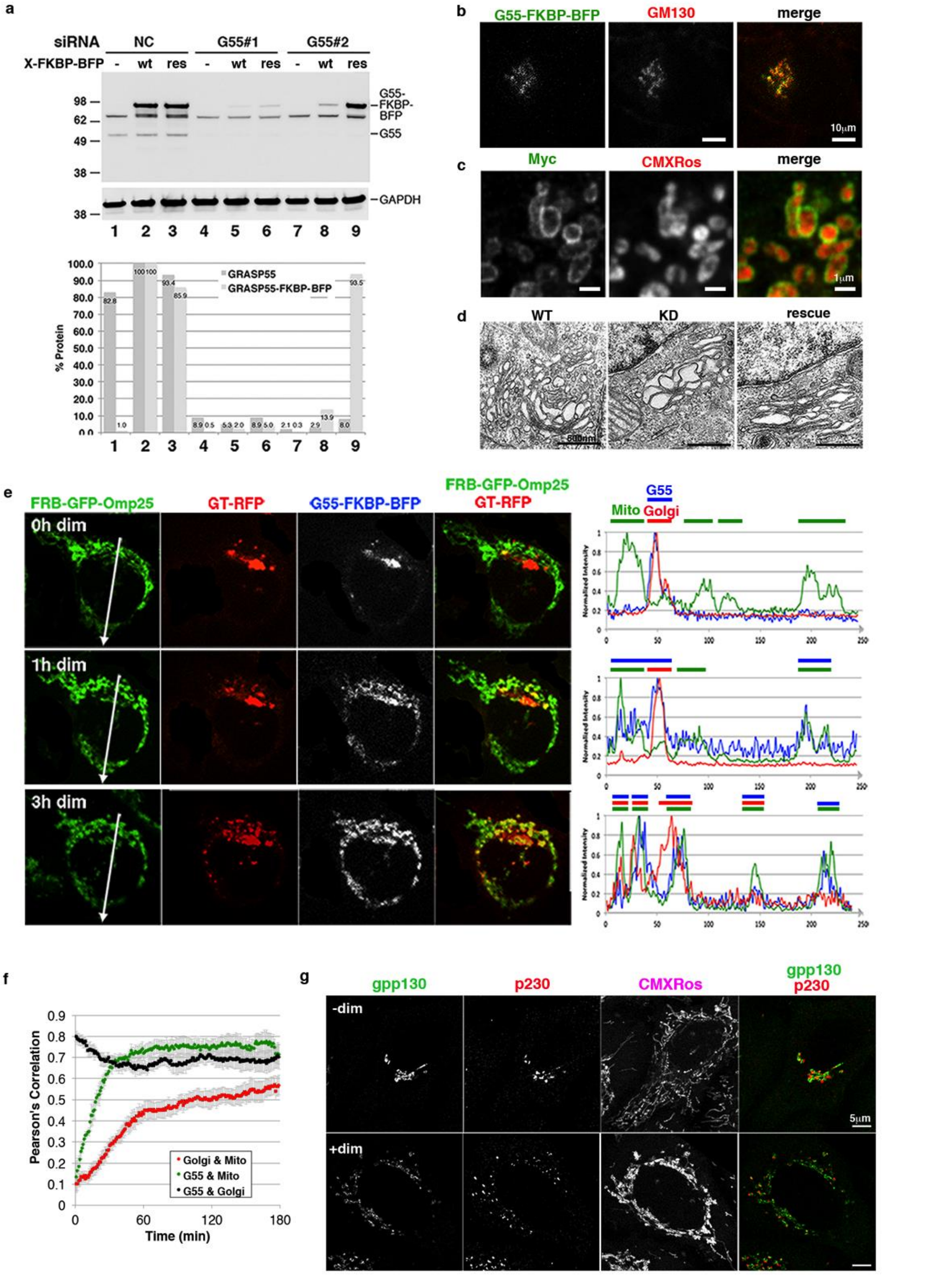

Supplementary Figure 1. Validating the GRASP55 re-routing assay. (**a**) Western blot analysis of HeLa cells treated with siRNA against negative control (NC), GRASP55#1, or

GRASP55#2 siRNA for 72 h, then transfected for 18 h with X-FKBP-BFP where X is empty (-), wild-type GRASP55 (wt), or siRNA-resistant GRASP55 (res). The membrane was immunoblotted for GRASP55 as well as GAPDH as a loading control. Quantification is shown below the blot. (**b**) Confocal image of HeLa cell transfected with GRASP55-FKBP-BFP and immunostained for GM130 (Golgi). Scale bar is 10 μm. (**c**) Confocal image of HeLa cell transfected with FRB-Myc-OMP25, stained with CMXRos (mitochondria) and immunostained for Myc. Scale bar is 1 μm. (**d**) TEM images of Golgi in wild-type, GRASP55 siRNA knockdown, and GRASP55-FKBP-BFP rescued HeLa cells. Scale bar is 500 nm. (**e**) Live imaging of GRASP55 re-routing assay. HeLa cells transfected with FRB-GFP-OMP25, GRASP55-FKBP-BFP, and GalT-RFP were imaged immediately after adding dimerizer. Frames from t = 0, 1 h, and 3 h are shown. A line profile was taken along the white line and displayed on the right to show the increased overlap of GRASP55, mitochondria and Golgi over time. (**f**) Pearson's Correlation Coefficient of GalT-RFP and FRB-GFP-OMP25 (Golgi & Mito, red), GRASP55-FKBP-BFP and FRB-GFP-OMP25 (G55 & Mito, green), and GRASP55-FKBP-BFP and GalT-RFP (G55 & Golgi, black) are plotted as a function of time (n = 5). Error bar = s.d. (**g**) Confocal images of HeLa cells transfected with GRASP55-FKBP-BFP and FRB-Myc-OMP25, then treated with 2 μM dimerizer for 3 h. Samples were stained with CMXRos (mitochondria), fixed, then immunostained for *cis*-Golgi (gpp130) and *trans*-Golgi (p230) markers. Scale bar is 5 μm.

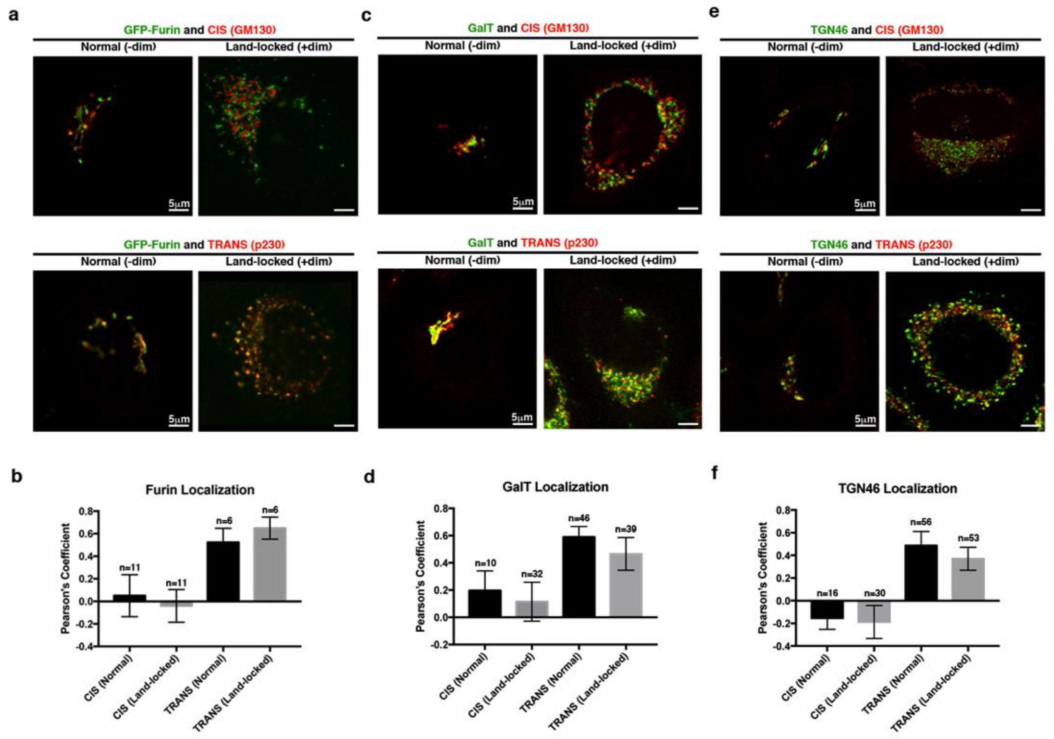

Supplementary Figure 2. Retargeting GRASP55 to mitochondria does not mislocalize Golgi markers. (**a**) Localization of Furin in land-locked Golgi cells. HeLa Cells were transfected with GFP-Furin, as well as GRASP55-FKBP-HA and FRB-Myc-Omp25, treated with dimerizer, then immunostained against GM130 (*cis*-Golgi) or p230 (*trans*-Golgi). Scale bar is 5 μm. (**b**) Pearson's Correlation Coefficient was calculated for GFP-Furin with GM130 and p230 in normal cells (no dimerizer) and land-locked Golgi cells (dimerizer). (**c**) Localization of GalT in land-locked Golgi cells. HeLa Cells were transfected with GRASP55-FKBP-GFP and FRB-Myc-Omp25, treated with dimerizer, then immunostained against GalT and GM130 (*cis*-Golgi) or GalT and p230 (*trans*-Golgi). Scale bar is 5 μm. (**d**) Pearson's Correlation Coefficient was calculated for GalT with GM130 and p230 in normal cells (no dimerizer) and land-locked Golgi cells (dimerizer). (**e**) Localization of TGN46 in land-locked Golgi cells. HeLa Cells were transfected with GRASP55-FKBP-GFP and FRB-Myc-Omp25, treated with dimerizer,

then immunostained against GM130 (*cis*-Golgi) or p230 (*trans*-Golgi). Scale bar is 5 μm. (**f**) Pearson's Correlation Coefficient was calculated for TGN46 with GM130 and p230 in normal cells (no dimerizer) and land-locked Golgi cells (dimerizer).

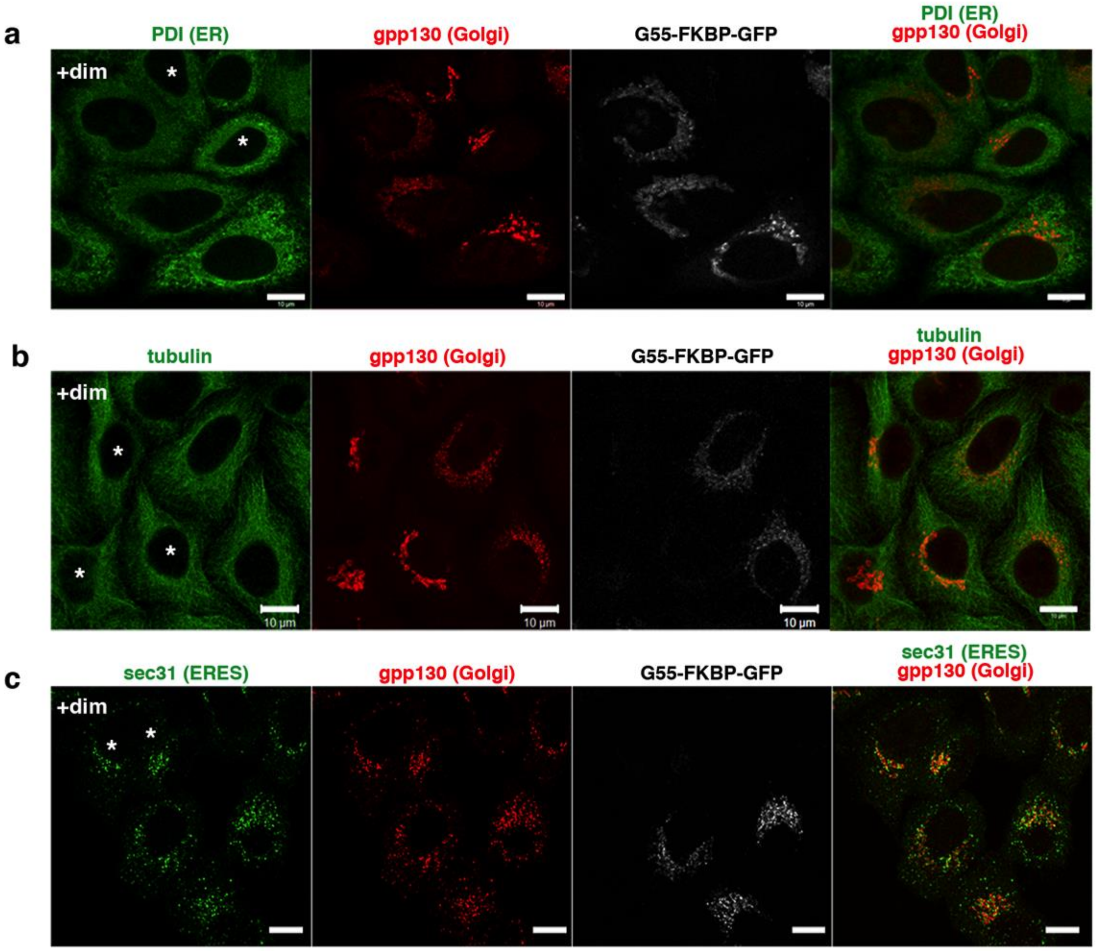

Supplementary Figure 3. Re-targeting GRASP55 to mitochondria does not disrupt ER and microtubule, but reorganizes ERESs. (**a**) Confocal images of HeLa cells transfected with GRASP55-FKBP-GFP and FRB-Myc-OMP25, then treated with dimerizer and immunostained for PDI (ER) and gpp130 (Golgi). Untransfected cells are labeled with an asterisk for comparison. (**b**) Same as (a), but cells were immunostained for α-tubulin and gpp130 (Golgi). (**c**) Same as (a), but cells were immunostained for sec31 (ERES, ER exit site) and gpp130 (Golgi). Scale bar is 10 μm.

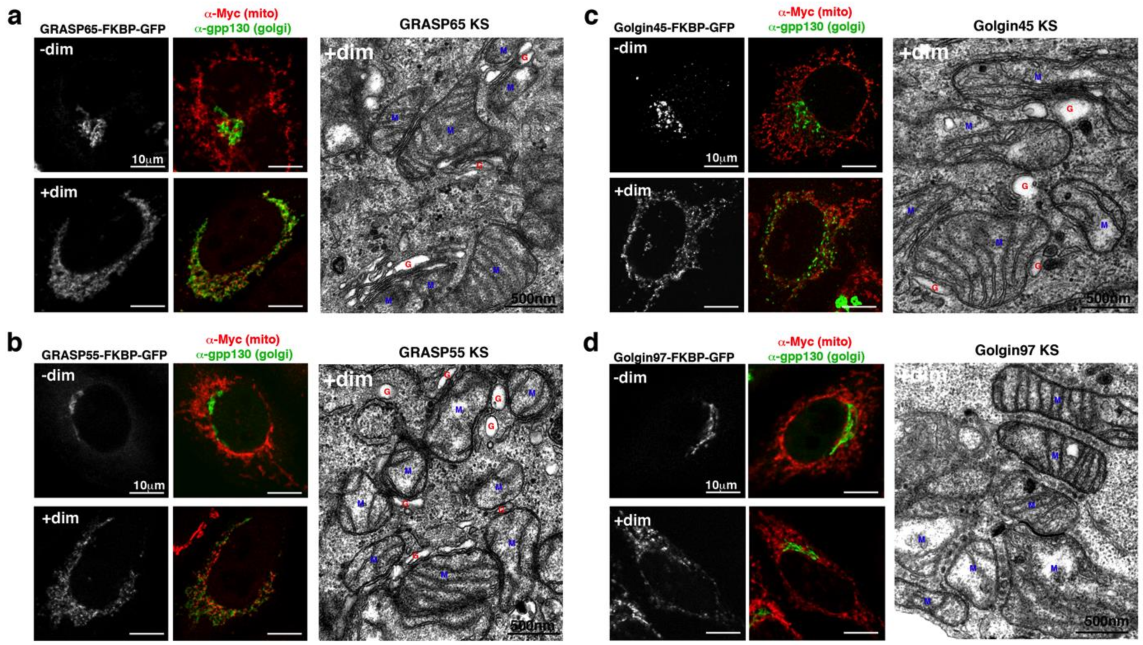

Supplementary Figure 4. Retargeting other Golgi proteins to the mitochondria. (**a-d**) Confocal and TEM images of HeLa cells transfected with X-FKBP-GFP and FRB-Myc-OMP25, then treated with 2 μM dimerizer for 3 h before fixation. For confocal images, cells were immunostained for gpp130 (Golgi) and Myc (mitochondria) antibodies. Scale bar is 10 μm. For EM images, Golgi are marked with a red G and mitochondria are marked with a blue M. Scale bar is 500 nm. (**a**) X = GRASP65, (**b**) X = GRASP55 (control), (**c**) X = Golgin45, (**d**) X = Golgin97.

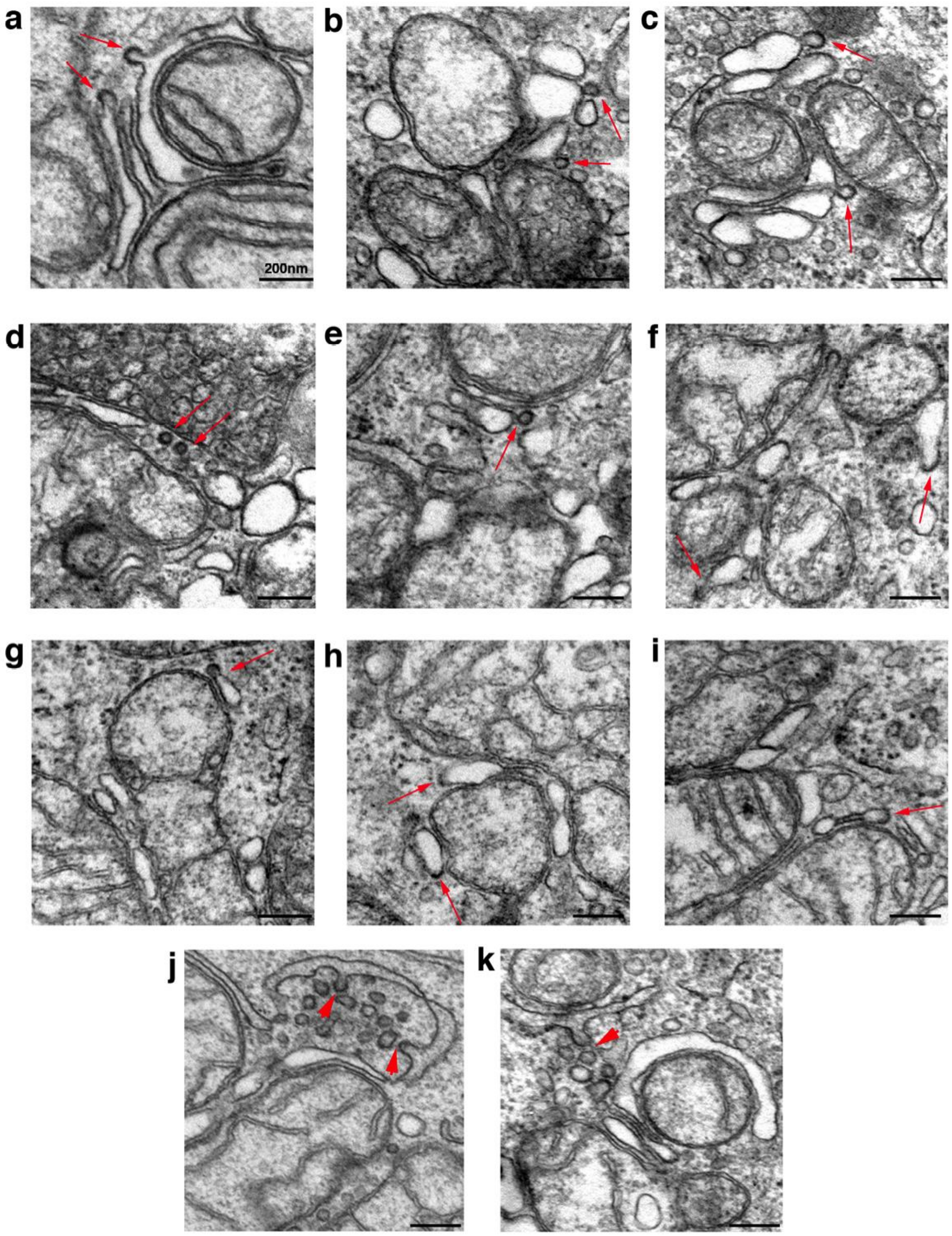

Supplementary Figure 5. EM gallery of vesicle budding regions on land-locked Golgi. (**a-i**) TEM images of presumptive COPI vesicles budding from land-locked Golgi

membranes. (**j-k**) TEM images of presumptive COPII vesicles budding from ER to nearby land-locked Golgi. Scale bar is 200 nm.

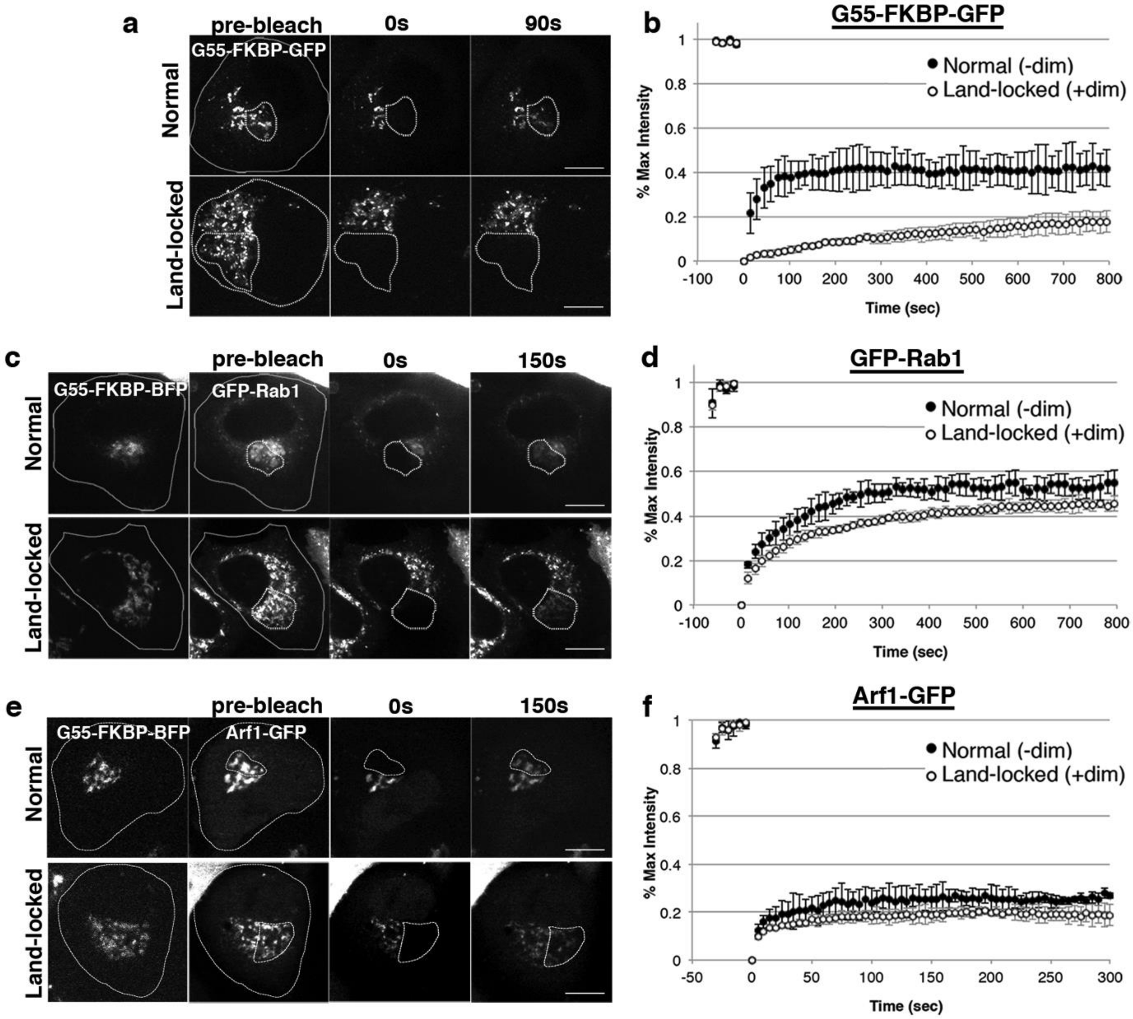

Supplementary Figure 6. Vesicle Budding machinery dynamically cycles on and off land-locked Golgi membranes. Fluorescence Recovery After Photobleaching (FRAP) was performed on GRASP55-FKBP-GFP/BFP and FRB-Myc-OMP25 transfected HeLa cells that were pre-treated with dimerizer for 3 h. Half of the Golgi area was bleached, then monitored for fluorescence recovery. (**a**) Confocal images of FRAP with GRASP55-FKBP-GFP. (**b**) Quantification of GRASP55-FKBP-GFP fluorescence in region of interest in (a), n = 3. (**c**) Confocal images of FRAP with GFP-Rab1. (**d**) Quantification of GFP-Rab1 fluorescence in region of interest in (c), n = 4. (**e**) Confocal

images of FRAP with Arf1-GFP. (**f**) Quantification of Arf1-GFP fluorescence in region of interest in (e), n = 3. Scale bar is 20 μm. Error bar = s.d.

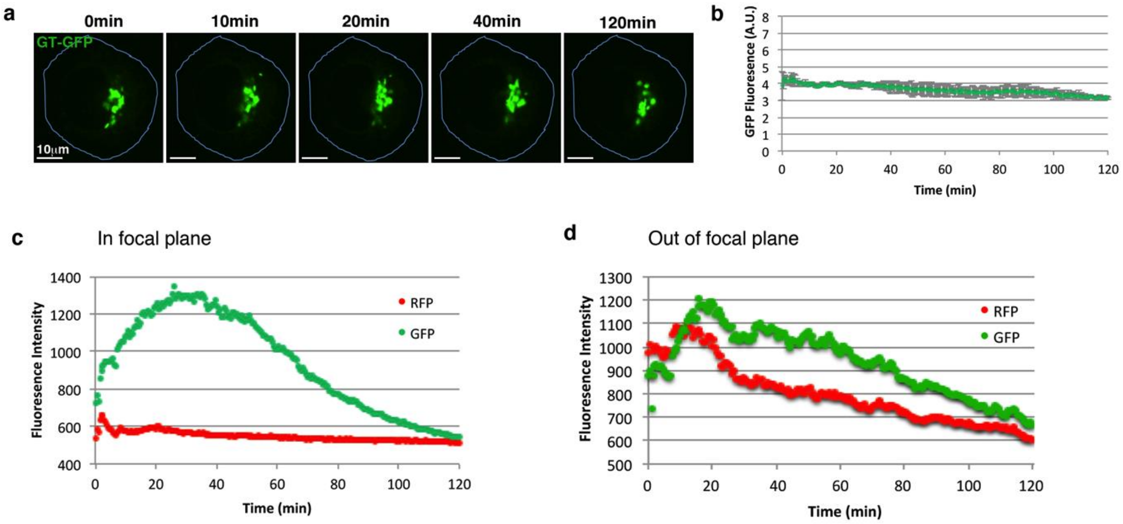

Supplementary Figure 7. Testing for GFP photobleaching and Selecting movies without drift. (**a**) HeLa cells were transfected with GalT-GFP, then imaged 30 s per frame for 2 h. Scale bar is 10 μm. (**b**) Quantification of GFP fluorescence at the Golgi in (a). (**c-d**) Both ssGFP-FM4-FCS-hGH and GalT-RFP were monitored in our trafficking assays. (**c**) We assumed that the focal plane did not drift if the red fluorescence signal remained constant in the Golgi area throughout the imaging time, even as the green fluorescence signal peaked and waned. These movies were selected for quantification. (**d**) However, if the red fluorescence signal decreased with the green fluorescence signal in the Golgi area during the course of the imaging time, then we assumed that the focal plane had drifted and did not include these movies for analysis.

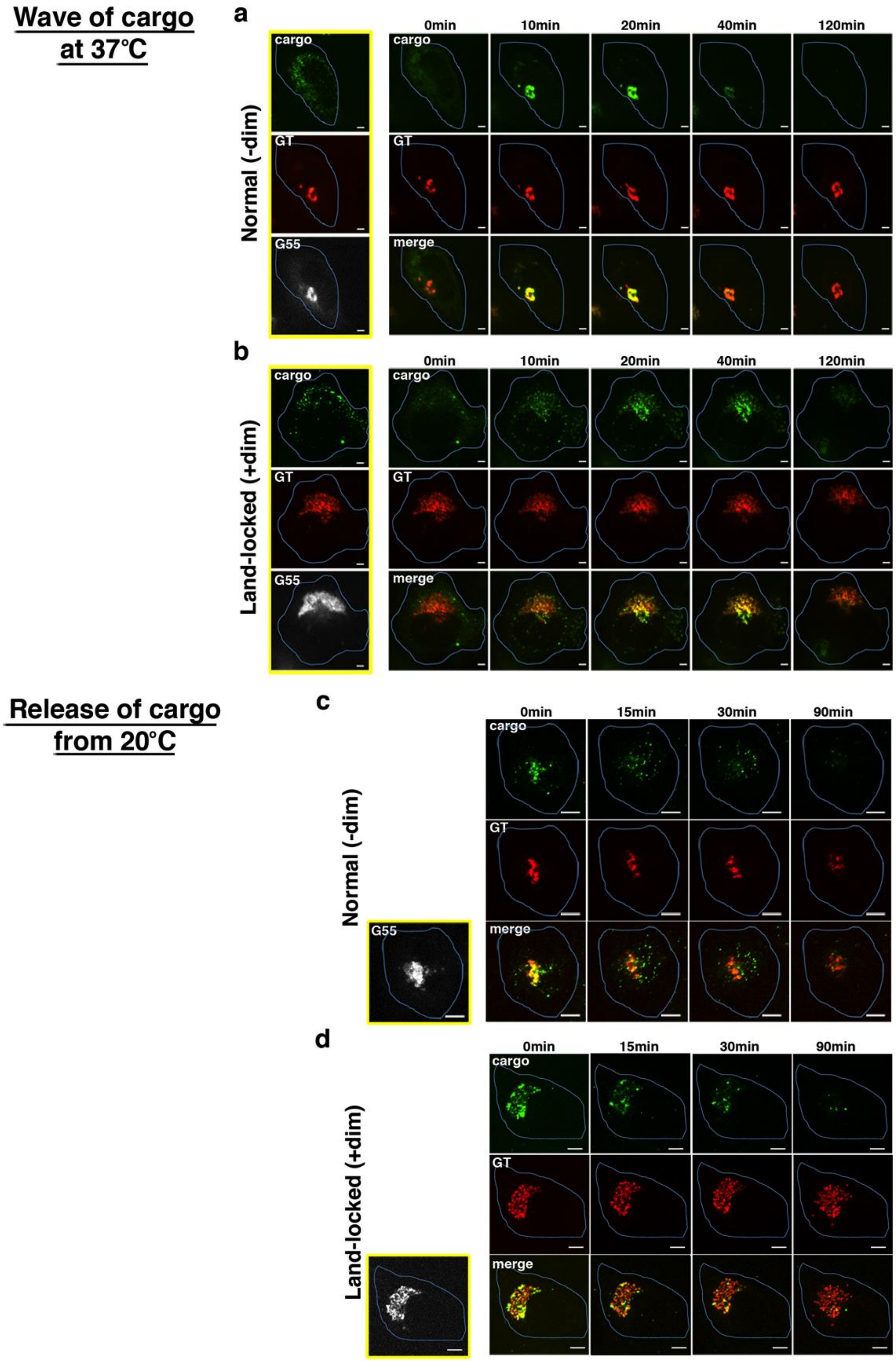
Wave of cargo at 37°C
a
0min
10min
20min
40min
120min
cargo
GT
G55
merge
Normal (-dim)
b
Land-locked (+dim)
Release of cargo from 20°C
c
0min
15min
30min
90min
d

Supplementary Figure 8. Live imaging wave of cargo in land-locked Golgi. (**a-b**) Live imaging of ssGFP-FM4-FCS-hGH traffic in HT1080 cells that stably express ss-GFP-FM4-FCS-hGH cargo and transfected with GRASP55-FKBP-BFP, FRB-Myc-OMP25, and GalT-RFP. (**a**) Normal Golgi (no dimerizer) and (**b**) land-locked Golgi (3 h dimerizer pre-treatment) were imaged at 30 s per frame for 2 h after adding solubilizer in the presence of cycloheximide. (**c-d**) Same as (a-b), except cells were cooled to 20°C for 1 h before adding solubilizer for 2 h, then warmed back to 37°C to begin imaging cargo exit from the TGN. Scale bar is 10 μm.

Wave of cargo at 37°C

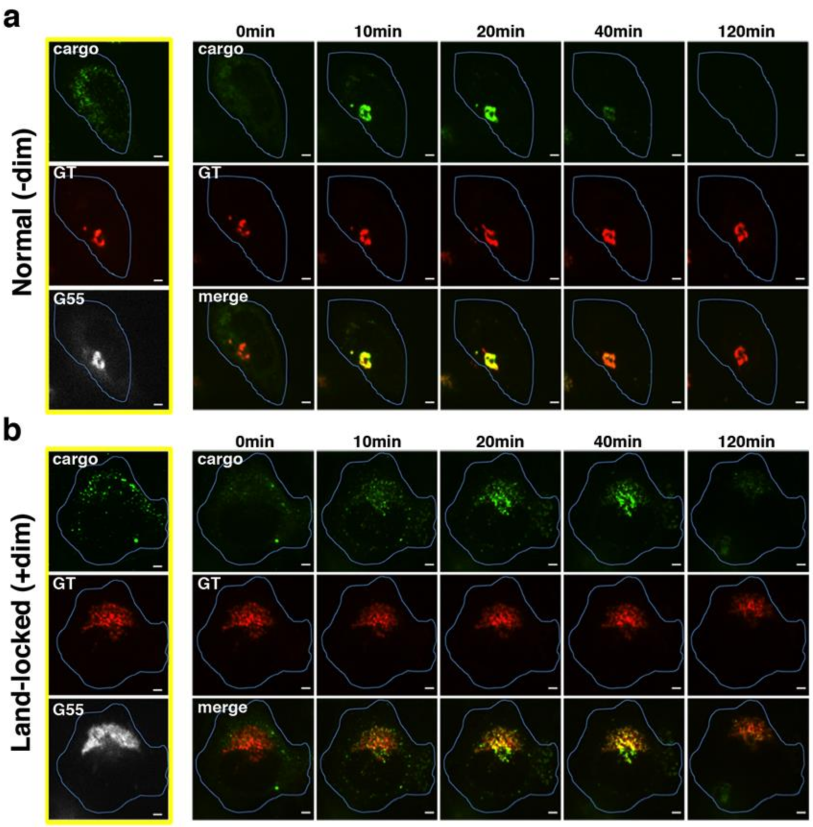

Release of cargo from 20°C

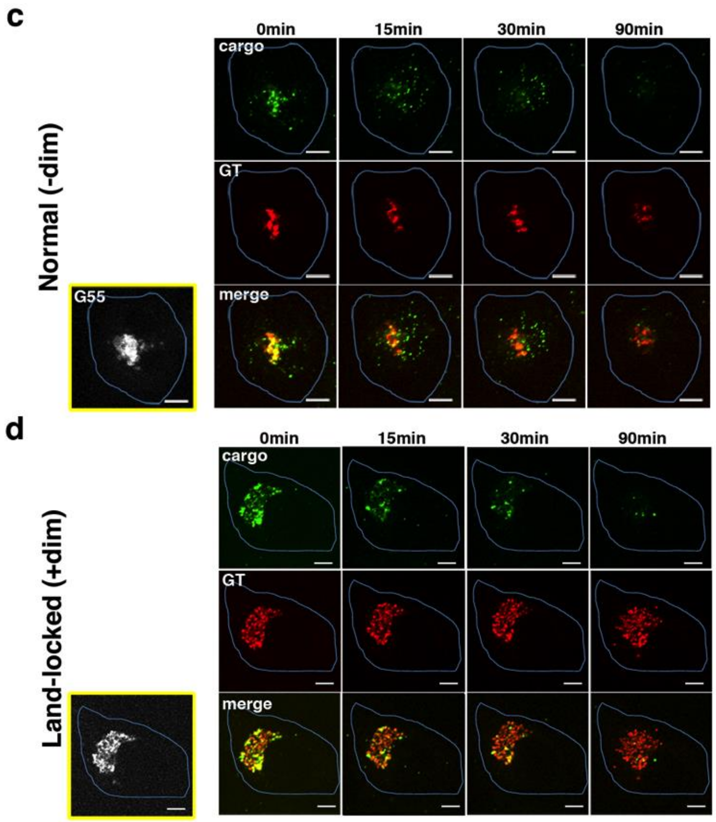

Supplementary Figure 9. Wave of cargo through individual normal or land-locked Golgi areas, imaged at 5 min per frame. Live imaging a wave of cargo at 37°C with HT1080 cells that stably express the ss-GFP-FM4-FCS-hGH cargo and transfected GRASP55-FKBP-BFP, FRB-Myc-OMP25, and GalT-RFP. (**a**) Normal Golgi (no dimerizer) and (**b**) land-locked Golgi (3 h dimerizer pretreatment) were imaged at 5 min per frame for 2 h after adding solubilizer in the presence of cycloheximide. (**c**) Normalized GFP fluorescence at the Golgi was quantified. (**d**) Traffic waves (mean) from Fig. 4c and Supplementary Fig. 7c (acquired at different imaging rates) are superimposed to show their similar rates, indicating that GFP photobleaching (if any occurs) does not influence the quantification. Scale bar is 10 μm. Error bars = s.d.

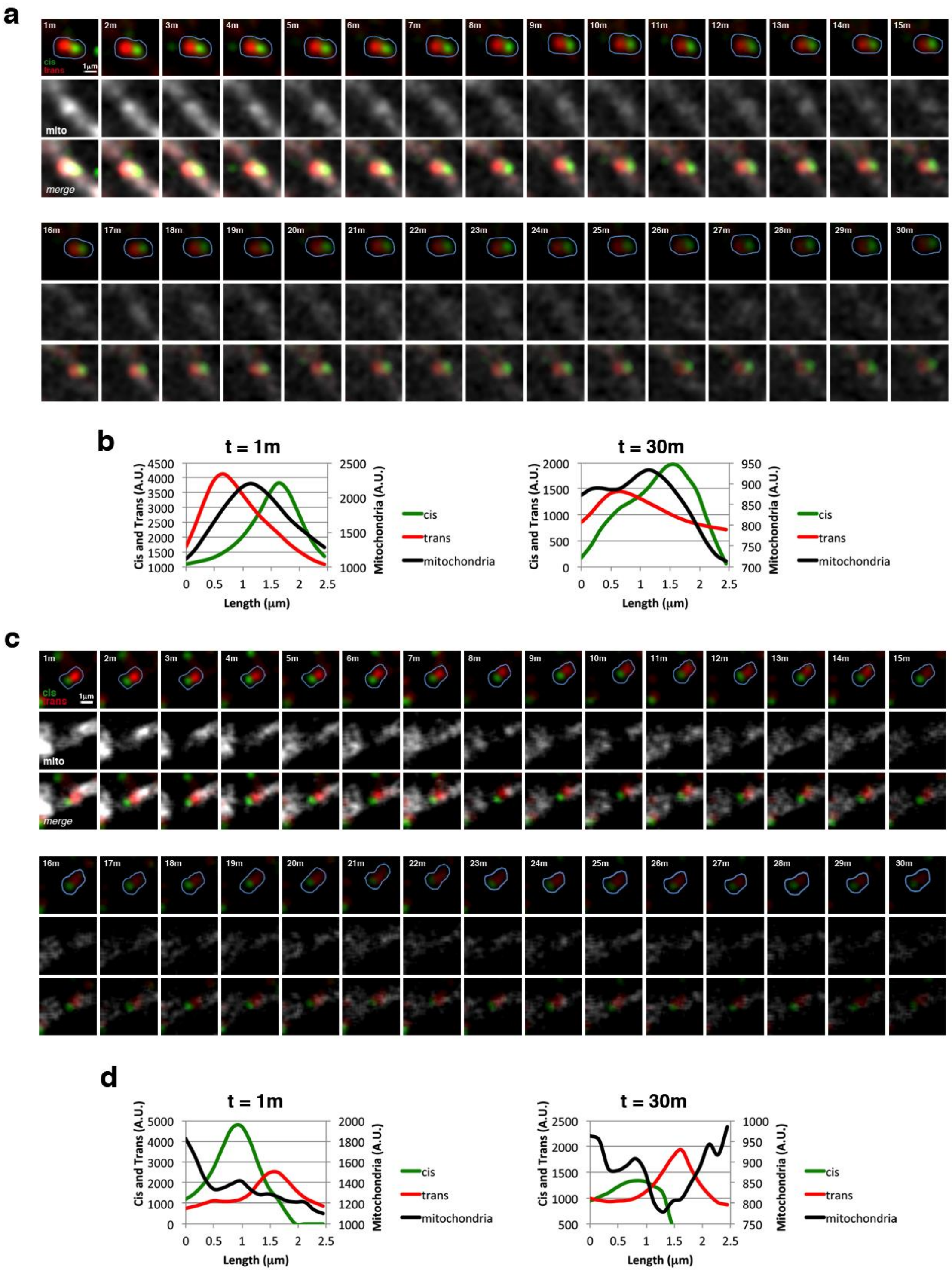

Supplementary Figure 10. More examples of live imaging land-locked Golgi. HeLa cells were transfected with G55-FKBP-BFP and FRB-Myc-Omp25 to be able to reroute

Golgi to the mitochondria, as well as GRASP65-GFP to mark the *cis*-Golgi and GalT-RFP to mark the *trans*-Golgi. Cells were treated with nocodazole for 3 h to break the Golgi ribbon into mini-stacks, and then dimerizer for 3 h to produce land-locked Golgi. Then they were imaged every minute in 3-D for 30 min. Two land-locked Golgi are shown in **(a)** and **(c)**, and their plot profiles are shown in **(b)** and **(d).** Scale bar = 1 μm.

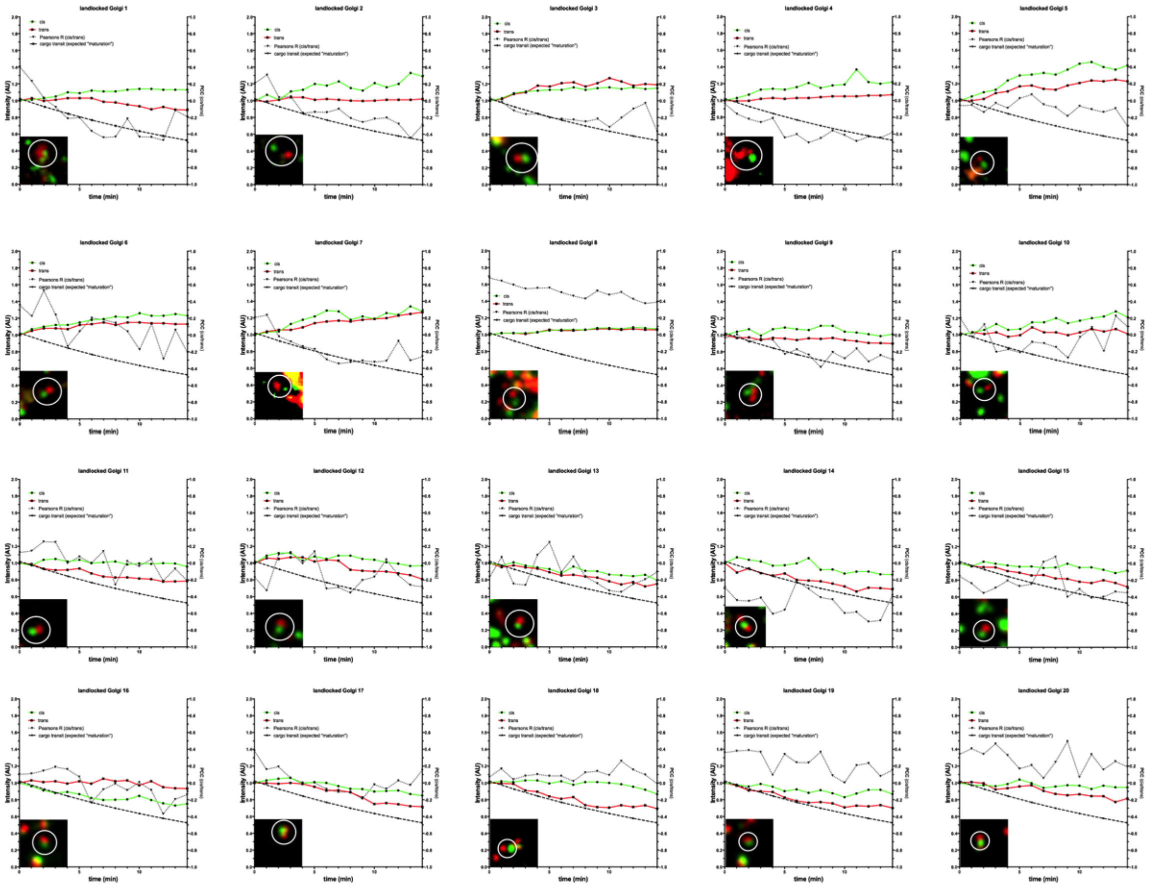

Supplementary Figure 11. Gallery of tracked landlocked Golgi cisternae. HeLa cells were transfected with GRASP55-FKBP-BFP and FRB-Myc-Omp25 to be able to re-target Golgi to the mitochondria, as well as GRASP65-GFP to mark the *cis*-Golgi and GalT-RFP to mark the *trans*-Golgi. Individual juxtaposed, yet separated and land-locked *cis* and *trans*-Golgi cisternae were tracked over 15 min using merged planes per time point (n=20, a visual correlate for each tracked example is given, highlighted by a white circle). The inversed rate of hGH arrival at the *trans*-Golgi (Fig. 5e) is plotted for comparison (expected rate of maturation; dashed line, left y-axis). Dotted line: Pearson´s correlation coefficient (PCC) for the tracked pair is given (right y-axis). The integrated intensity of the *cis* and *trans*-Golgi markers was tracked over time and normalized to initial intensity (red and green lines, left y-axis). Land-locked Golgi 1-10 were corrected

to mitochondria marker G55-BFP due to a drift in the experiment, land-locked Golgi 11-20 represent raw data tracks.

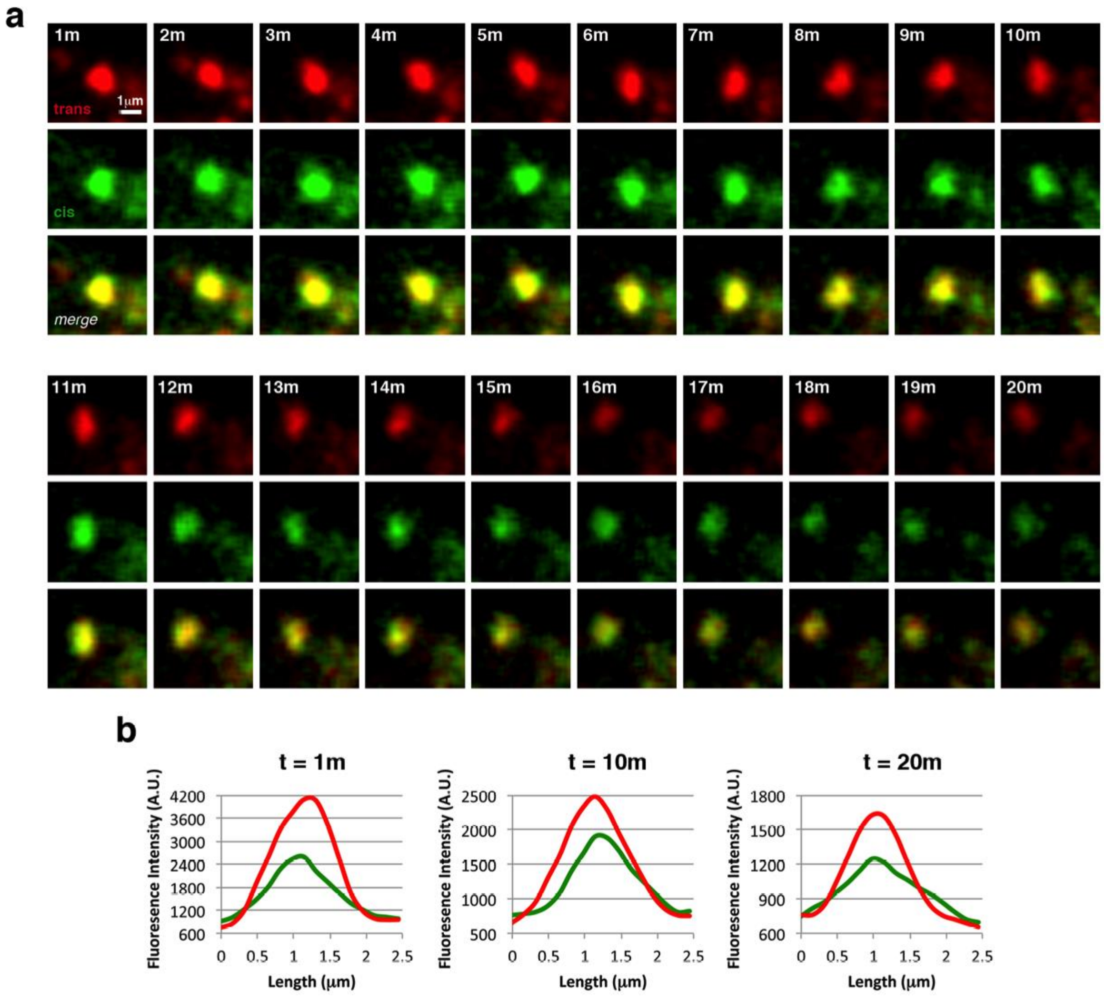

Supplementary Figure 12. *Cis*- and *trans*-Golgi cisternae cannot be easily resolved by confocal microscopy. (a) HeLa cells were transfected with G55-FKBP-BFP and FRB-Myc-Omp25, as well as GRASP65-GFP to mark the *cis*-Golgi and GalT-RFP to mark the *trans*-Golgi. Cells were treated with nocodazole for 3 h to break the Golgi ribbon into mini-stacks, but no dimerizer was added. Then they were imaged every minute in 3-D for 20 min. **(b)** Plot profiles were taken for the mini-stack shown in (a) for time points 1 min, 10 min, and 20 min.
